# Molecular Basic of Pharmacotherapy of Cytokine Imbalance as a Component of Intervertebral Disc Degeneration Treatment

**DOI:** 10.3390/ijms24097692

**Published:** 2023-04-22

**Authors:** Natalia A. Shnayder, Azamat V. Ashkhotov, Vera V. Trefilova, Zaitun A. Nurgaliev, Maxim A. Novitsky, Marina M. Petrova, Ekaterina A. Narodova, Mustafa Al-Zamil, Galina A. Chumakova, Natalia P. Garganeeva, Regina F. Nasyrova

**Affiliations:** 1 Institute of Personalized Psychiatry and Neurology, Shared Core Facilities, V.M. Bekhterev National Medical Research Centre for Psychiatry and Neurology, 192019 Saint Petersburg, Russia; ashkhotov.v@mail.ru (A.V.A.); zaitun97@mail.ru (Z.A.N.); 2 Shared Core Facilities “Molecular and Cell Technologies”, V.F. Voino-Yasenetsky Krasnoyarsk State Medical University, 660022 Krasnoyarsk, Russia; stk99@yandex.ru (M.M.P.); katya_n2001@mail.ru (E.A.N.); 3 Department of Neurology, Hospital for War Veterans, 193079 Saint Petersburg, Russia; vera.v.trefilova@yandex.ru (V.V.T.); maximnovitsky93@gmail.com (M.A.N.); 4 Department of Physiotherapy, Faculty of Continuing Medical Education, Peoples’ Friendship University of Russia, 117198 Moscow, Russia; alzamil@mail.ru; 5 Department of Therapy and General Medical Practice with a Course of Postgraduate Professional Education, Altai State Medical University, 656038 Barnaul, Russia; g.a.chumakova@mail.ru; 6 Department of General Medical Practice and Outpatient Therapy, Siberian State Medical University, 634050 Tomsk, Russia; garganeeva@gmail.com; 7 International Centre for Education and Research in Neuropsychiatry, Samara State Medical University, 443016 Samara, Russia

**Keywords:** cytokine, cytokine status, chronic inflammation, intervertebral disc degeneration, molecular mechanism, treatment

## Abstract

Intervertebral disc degeneration (IDD) and associated conditions are an important problem in modern medicine. The onset of IDD may be in childhood and adolescence in patients with a genetic predisposition. With age, IDD progresses, leading to spondylosis, spondylarthrosis, herniated disc, spinal canal stenosis. One of the leading mechanisms in the development of IDD and chronic back pain is an imbalance between pro-inflammatory and anti-inflammatory cytokines. However, classical therapeutic strategies for correcting cytokine imbalance in IDD do not give the expected response in more than half of the cases. The purpose of this review is to update knowledge about new and promising therapeutic strategies based on the correction of the molecular mechanisms of cytokine imbalance in patients with IDD. This review demonstrates that knowledge of the molecular mechanisms of the imbalance between pro-inflammatory and anti-inflammatory cytokines may be a new key to finding more effective drugs for the treatment of IDD in the setting of acute and chronic inflammation.

## 1. Introduction

Intervertebral disc degeneration (IDD) is an urgent public health problem. The main manifestation of IDD is acute and chronic back pain. It is experienced by more than 85% of people over 35 years of age [1]. Discogenic back pain is the leading cause of disability benefits in the social security system [2]. IDD is associated with damage to nearby ligaments, joints, and paravertebral muscles. Degenerated intervertebral discs (IVDs) are located lower than normal, and the apophyseal joints bear higher loads [3]. The consequence of this is osteoarthritis degeneration. The strength of the yellow ligaments decreases, which leads to their hypertrophy and protrusion of the ligaments into the spinal canal, followed by narrowing and compression of the neural structures [4].

**Figure 1:**
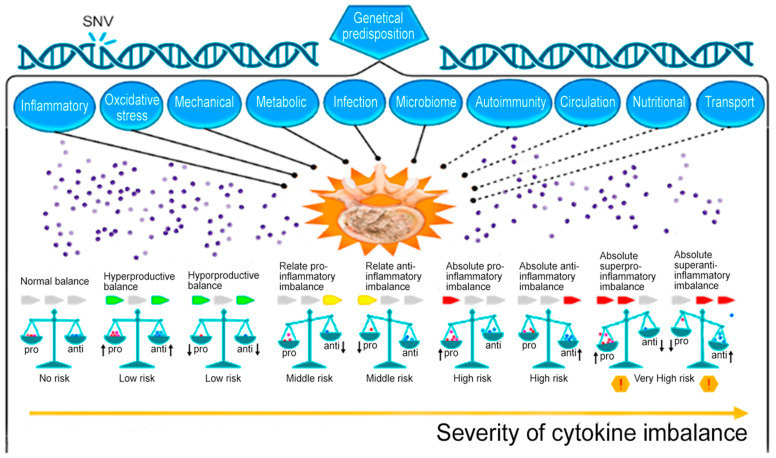
Potential role of normal and abnormal cytokine levels in the cytokine imbalance as a biomarker of intervertebral disc degeneration. Note: SNV—single-nucleotide variant; pro—proinflammation cytokines; anti—anti-inflammation cytokines. Note: solid line—well-studied mechanisms of IDD development; dotted line—insufficiently studied mechanisms of IDD development; up arrow—increased cytokine level; down arrow—decreased cytokine level; red exclamation point in a yellow hexagon is a dangerous situation [6].

The pathogenesis of acute and chronic pain in IDD in complex cases represents a combination of structural and mechanical deformities, as well as an increase in the activity of inflammatory mediators, including cytokines [5]. The most unfavorable in terms of the rate of progression of IDD and the formation of vertebrogenic pain syndrome is an absolute high-producing cytokine imbalance (Figure 1) [6]. This indicates that a simple measurement of one or more pro-inflammatory cytokines in the blood is not enough to make a final conclusion about the nature of changes in the cytokine balance in a patient with IDD, as well as to make an informed decision on the choice of a therapeutic strategy. for the purpose of its correction [6].

Knowledge of the molecular mechanisms of cytokine imbalance, as one of the clinically significant pathogenetic mechanisms for the development of IDD, can help in the search for promising effective therapeutic strategies to prevent the progression of degenerative processes in the IVD and surrounding tissues, as well as to prevent or reduce the degree of involvement of neighboring spinal motion segments into the pathological process. This fundamental approach may improve the expected therapeutic response to existing classical and emerging drug treatments for IDD. 

The purpose of this narrative review is to update knowledge about new and promising therapeutic strategies based on the correction of molecular mechanisms of cytokine imbalance in patients with IDD.

## 2. General Approaches to Intervertebral Disc Degeneration Therapy

**Figure 2:**
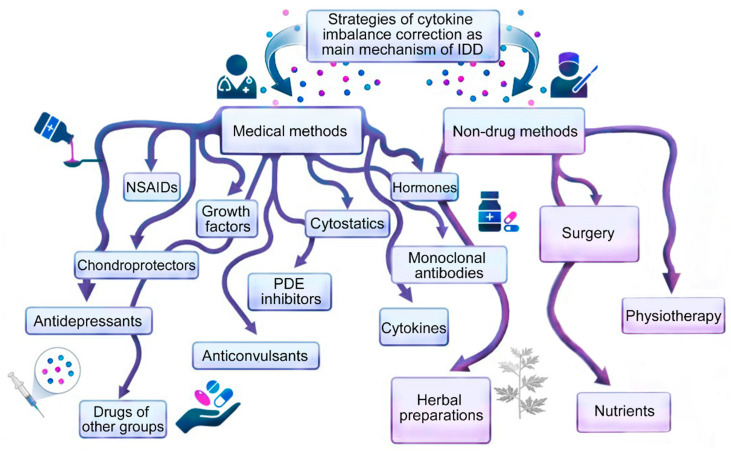
Methods for correcting cytokine imbalance in patients with intervertebral disc degeneration.

General (classical) approaches to the treatment of cytokine imbalance in patients with IDD (Figure 2) are based primarily on the symptomatic correction of inflammation and pain with systemic drugs or focal injections into the inflamed degenerating IVD or direct surgical removal of damaged IVD tissue [7]. Non-drug therapies such as exercise, weight loss, and physiotherapy are used for IDD, but only when the IDD is not severe [8]. Current treatments for IDD in sequenced or non-sequenced IVD hernias range from lifestyle changes to invasive surgical interventions, as the causes of IDD are diverse (professional habits, lifestyle, smoking, etc.) and depend on age, genetic predisposition and cellular -mediated degradation reactions accelerating after traumatic or prolonged mechanical impacts, leading to the abnormal production of cytokines and catabolic molecules by cells of degenerating IVD [9,10,11].

It is possible that an inflammatory response is involved in the onset of the disease, but it is also critical for maintaining tissue homeostasis in degenerating IVDs. In addition, optimal cytokine balance may contribute to tissue repair/regeneration of damaged IVDs [12]. Traditionally, the inflammatory response has been viewed as a negative injury mechanism that correlates with the progression of IDD. However, it remains unclear whether chronic inflammatory response in general, and cytokine imbalance in particular, is a cause or consequence of the development of IDD and herniation of IVDs. It is likely that a balanced inflammatory response is needed to reduce the rate of progression of IDD and reduce the severity of symptoms of the disease, as well as for the most complete (most possible in each specific clinical case) restoration of the function of damaged IVDs. The delicate balance between pro-inflammatory and anti-inflammatory cytokines largely determines the overall effect of the inflammatory response in patients with IDD and the expected therapeutic response to prescribed drugs. Cytokine imbalance may direct the protective immune response towards chronic inflammation (pro-inflammatory effect) or healing (anti-inflammatory effect) in patients with IDD. On the one hand, cytokine imbalance may be beneficial for IDD patients by initiating an inflammatory response in the nucleus pulposus (NP), annulus fibrosis (AF), and extracellular matrix of IVDs. On the other hand, the excessive or insufficient production of pro-inflammatory or anti-inflammatory cytokines can be harmful and initiate the development and progression of IDD [6].

The study of the molecular basis of inflammatory responses in the complex spatial and temporal organization of cellular interactions in the nucleus pulposus (NP) and annulus fibrosus (AF), as well as in the remodeling of the extracellular matrix of IVDs, can play an important role in the search for effective methods of IDD pharmacotherapy, since many of the existing therapeutic strategies do not yet achieve the expected therapeutic response [6].

## 3. Medication Methods for Correcting Cytokine Imbalance

General (classical) and new (promising) methods of pharmacotherapy of cytokine imbalance in patients with IDD are summarized by us.

### 3.1. Nonsteroidal Anti-Inflammatory Drugs

#### 3.1.1. Salicylates

Acetylsalicylic acid (ASA)-2-(acetyloxy)benzoic acid) is a salicylate used to relieve pain, fever, and inflammation, including those associated with IDD. ASA disrupts the production of prostaglandins throughout the body, affecting cyclooxygenase-1 (COX-1) and cyclooxygenase-2 (COX-2) [13]. By disrupting the production and preventing the release of prostaglandins during inflammation, this drug can stop their action on pain receptors, substances such as histamine and bradykinin [14]. Additionally, ASA is involved in the regulation of cytokine balance. The protective mechanism of ASA includes the activation of the 5′ adenosine monophosphate (AMP)-activated protein kinase (AMPK) signaling pathway, which attenuates oxidative stress. AMPK mediates energy metabolism at the intracellular level and can suppress various pathological oxidative stressors or inflammatory responses in IVD [15]. The activity of the AMPK signaling pathway leads to a significant decrease in the production of oxidative stressors, including reactive oxygen species (ROS) and inducible nitric oxide synthase (iNOS) [16,17]. In a study by Liu et al. [18], ASA significantly reduced iNOS functional activity and NO synthesis, indicating that it attenuates oxidative stress in IDD. Reducing NO production by inhibiting iNOS can significantly reduce the production of matrix metalloproteinases (MMPs) and interleukin 1 beta (IL-1β) [19]. Tsoyi et al. [20] and Chen et al. [21] concluded that an increase in iNOS expression leads to an increase in NO synthesis, while AMPK activation attenuates iNOS expression in NP. However, this contradicts the results of Lee et al. [22], who found that AMPK promotes iNOS expression and NO production in epithelial cells of the proximal tubules of the kidneys. These results indicated that iNOS is a positive factor protecting IVD cells from oxidative stress. However, iNOS inhibition does not slow the progression of osteoarthritis in humans. Liu et al. [18] suggest that the pathogenetic mechanisms of osteoarthritis may differ from those of IDD, and iNOS may play different physiological roles in different cell lines.

Mesalazine (5-amino-2-hydroxybenzoic acid) is an aminosalicylate drug. Mesalazine reduces inflammation by blocking COX-1 and COX-2 and inhibits the production of prostaglandins. Mesalazine has a pharmacodynamic effect due to direct activation of γ-receptors that activate peroxisome proliferation (PPAR) [23,24]. In addition, mesalazine is involved in the regulation of cytokine balance in another way. For example, it can inhibit the activation of nuclear factor kappa B (NF-κB) and hence the production of key pro-inflammatory cytokines [23,24]. Other studies have shown that mesalazine can inhibit iNOS expression [25]. However, the use of this drug in IDD has not been studied. Basically, mesalazine is used in ulcerative colitis [14].

Sulfasalazine (2-Hydroxy-5-[[4-[(2-pyridynylamino)sulfonyl]phenyl]azo]benzoic acid) and its metabolites (e.g., sulfapyridine) can inhibit leukotrienes and prostaglandins by blocking the cyclooxygenase and lipoxygenase pathways of inflammation [26]. Additionally, the inhibitory activity of sulfosalazine was observed on arachidonic acid derivatives, including PPAR-γ [27]. In addition, this drug plays a role in the regulation of cytokine balance. Thus, sulfosalazine inhibits the activity of NF-κB and IkappaB kinases α and β [23,28], which play an important role in the NF-κB signaling pathway, which is activated by a variety of stimuli, including pro-inflammatory cytokines, bacterial or viral products, DNA damage, or others [14]. Sulfasalazine, similarly to mesalazine, has promise for correcting cytokine imbalances in patients with IDD and severe back pain, but so far it has been used in gastroenterology for ulcerative colitis.

#### 3.1.2. Pyrazolones

Edaravone (1-phenyl-3-methyl-5-pyrazolone) is an ROS scavenger and neuroprotective agent with antioxidant properties. Edaravone absorbs ROS, acting on oxidative stress, and also inhibits the formation of hydroxyl and peroxynitrite radicals [29]. However, the exact mechanism of action of this drug has not yet been elucidated. Although it has been shown that edavaron is involved in the regulation of cytokine balance. It is hypothesized that this drug may have anti-inflammatory properties as it inhibits neutrophil activation and downregulates iNOS and neuronal NOS (nNOS) expression in animal models. This leads to the suppression of the activity of pro-inflammatory cytokines. Additionally, it improves ROS-induced inflammatory oxidative stress [29]. However, the possibility of using edavaron to correct cytokine imbalance in IDD needs to be studied in the future.

#### 3.1.3. Derivatives of Organic Acids

Indomethacin (1-(p-chlorobenzoyl)25-methoxy-2-methylindole-3-acetic acid) has anti-inflammatory, analgesic, and antipyretic properties [30]. Indomethacin is a non-steroidal anti-inflammatory drug (NSAID), a non-selective COX inhibitor. In particular, indomethacin is a non-specific and reversible inhibitor of COX-2. Indomethacin, unlike other NSAIDs, also inhibits phospholipase A2, the enzyme responsible for the release of arachidonic acid from phospholipids [14]. Indomethacin has been shown to be involved in the regulation of cytokine balance in IDD patients as an adapter protein and/or through its kinase activity. It can regulate various signaling pathways (p38 MAP kinase, NF-κB and insulin signaling pathways) and transcription factors (JUN-transcription factor subunit AP-1 [activation protein 1], STAT1 [signal transducer and activator of transcription 1], STAT3 [signal transducer and activator of transcription 3]-signal transducers and transcription activators, IRF1 [interferon regulatory factor 1], ATF3 [activating transcription factor 3]) involved in the expression of genes encoding pro-inflammatory cytokines and interferons. Indomethacin activates the NF-κB pathway by interacting with proteins of the nuclear factor kappa-B beta-kinase β-kinase (IKBκB) inhibitor family and TRAF-interacting protein (TRAF), and it also activates the p38 mitogen-activated protein kinase (p38 MAP) pathway through interaction with mitogen-activated protein kinase 6 (MAP2K6). Indomethacin can regulate inflammasome assembly with the NLR family pyrine domain containing protein 3 (NLRP3) and inflammasome activation of NLRP3, NLRP1, melanoma non-existent protein 2 (AIM2), and cytosolic protein, a Nod-like receptor from the NOD subfamily (nucleotide-binding domain oligomerization receptors), also known as NLRC4 [31,32].

Ibuprofen ((RS)-2-(4-(2-methylpropyl)phenyl)propanoic acid) is an NSAID and is considered the first of the propionic acids in search of a safer alternative to ASA [33]. Ibuprofen is a non-selective COX inhibitor and therefore inhibits the activity of both COX-1 and COX-2. The inhibition of COX-2 activity reduces the synthesis of prostaglandins involved in mediating inflammation, pain, fever, and edema, while the inhibition of COX-1 is believed to cause some adverse drug reactions (ADRs) of ibuprofen [34]. Ibuprofen suppresses apoptosis in various cellular systems, including factor-dependent lymphohematopoietic and nerve cells (Apoptosis regulator Bcl-2) [35]. Additionally, ibuprofen is involved in the regulation of cytokine balance. In particular, it is effective in selectively inhibiting IL-8 chemotaxis in patients with IDD [36].

Ketoprofen (3-benzoyl-alpha-methylbenzeneacetic acid) is an NSAID. It is believed that the anti-inflammatory effect of ketoprofen is associated with the inhibition of COX-2. This leads to a decrease in the level of prostaglandins, which mediate pain, fever, and inflammation [14]. Ketoprofen is involved in the regulation of cytokine balance in patients with IDD. The results of the Bizzarri et al. [36] show that R- and S-ketoprofen, regardless of their activity as prostaglandin inhibitors, were very effective in selectively inhibiting IL-8 chemotaxis. The inhibition of IL-8 chemotaxis has been obtained at drug concentrations in excess of therapeutic plasma levels. The decrease in IL-8 migration by ketoprofen isomers was accompanied by the selective inhibition of the response of polymorphonuclear leukocytes due to an increase in the intracellular concentration of calcium ions and activation of kinase (ERK)-2, regulated by an extracellular signal. The results of Wang et al. [37] show that ketoprofen inhibits edema, suppresses capillary permeability, reduces IL-8 production, and increases superoxide dismutase activity in an acetic acid-induced inflammatory condition in an animal model, and therefore this drug is used in IDD and discogenic back pain.

Flurbiprofen (2-fluoro-alpha-methyl[1,1′-biphenyl]-4-acetic acid), like other NSAIDs, has an anti-inflammatory effect. This effect is manifested through a reversible inhibition of COX, which in turn leads to an effective decrease in the concentration of prostaglandins involved in inflammation, pain, edema, and fever. This drug is one of the most potent NSAIDs in terms of prostaglandin inhibitory activity [14]. Flurbiprofen may be involved in the regulation of cytokine balance in IDD. For example, flurbiprofen is effective in the selective inhibition of IL-8 chemotaxis [36]. However, its role in correcting cytokine imbalances in patients with IDD needs further study.

#### 3.1.4. Coxibs

Celecoxib (4-[5-(4-methylphenyl)-3-(trifluoromethyl)-1H-pyrazol-1-yl]benzenesulfonamide) is an NSAID and a selective noncompetitive inhibitor of the COX-2 enzyme [38]. The inhibition of this enzyme reduces the synthesis of metabolites, including prostaglandin E2, prostacyclin, thromboxane, prostaglandin D2, and prostaglandin F2 (PGF2). The resulting inhibition of these mediators leads to relief of pain and inflammation [39]. Celecoxib poses a lower risk of ulceration than other NSAIDs due to its lesser effect on prostaglandin synthesis in the gastric mucosa compared with placebo [40] but increases the risk of thromboembolic ADRs [14]. Additionally, celecoxib plays a role in the regulation of cytokine balance in IDD. It inhibits serine/threonine kinase, which negatively regulates TGF-β-induced signaling by: (1) modulating the association of SMAD-3 and SMAD-7 with the TGF-β receptor, and the phosphorylation of SMAD-2, SMAD-3, SMAD-4, and SMAD-7; (2) the prevention of nuclear translocation of SMAD-3 and SMAD-4, and translocation of SMAD-7 from the nucleus to the cytoplasm in response to TFR-β. Celecoxib activates the NF-κB pathway through IKκB phosphorylation, which is required for vascular endothelial cell motility and participation in the regulation of their chemotaxis. Additionally, celecoxib provides negative feedback inhibition of toll-like receptor-mediated NF-κB activation in macrophages [41,42].

#### 3.1.5. Derivatives of Indazole

Benzydamine (3-(1-benzyl-1H-indazol-3-yloxy)-N, N-dimethylpropan-1-amine) is an NSAID. Benzydamine is largely a weak inhibitor of prostaglandin synthesis, as it has been shown to effectively inhibit COX and lipoxygenase activity only at concentrations of 1 mM or higher. Benzydamine, due to the route of administration, often does not reach the degree of absorption or blood concentration necessary for the occurrence of any extraneous long-term systemic effects or inhibition of COX [43]. In addition, it is assumed that benzydamine is able to inhibit the oxidative burst of neutrophils and stabilize cell membranes by inhibiting the release of granules from neutrophils and stabilize lysosomes [43]. Benzydamine has a local anesthetic effect, which may be due to its ability to inhibit the release of inflammatory mediators, such as substance P and a peptide related to the calcitonin gene, from sensory nerve endings. Since substance P is able to induce the release of histamine from mast cells, preventing the release of substance P, benzydamine additionally contributes to the anti-inflammatory effect [43]. In addition, benzamide may be involved in the regulation of cytokine balance in IDD. This drug exhibits different mechanisms of action that differ from those of traditional ASA-like NSAIDs. In particular, benzydamine predominantly acts by inhibiting the synthesis of pro-inflammatory cytokines such as tumor necrosis factor alpha (TNF-α) and IL-1β, without significantly affecting other pro-inflammatory cytokines (such as IL-6 and IL-8) or anti-inflammatory cytokines (e.g., such as IL-10 or an IL-1 receptor antagonist) [44,45]. Benzydamine strongly inhibits ERK1/2 and p38 MAPK activation induced by chemotactic factors. However, MAPK inhibition is selective for chemoattractant-stimulated MAPK activation, since benzydamine does not affect lipopolysaccharide-stimulated activation [46].

### 3.2. Hormones

#### 3.2.1. Corticosteroids

Corticosteroids is the collective name for a subclass of steroid hormones produced exclusively by the adrenal cortex and having neither progestogenic, nor androgenic, nor estrogenic activity, but possessing either glucocorticoid or mineralocorticoid activity to one degree or another [47]. Intradiscal corticoid injections are performed to reduce intradiscal inflammation in patients with IDD and spinal nerve root injury caused by degenerated or sequenced NP [48]. However, only a 25% success rate has been shown with intradiscal steroids at short-term follow-up [49]. In the long term, this strategy has shown no clinical benefit in patients with chronic discogenic low back pain [50]. In addition, intradiscal corticosteroids are believed to help stabilize the spinal segment through further IDD [51]. The short-term effects of corticosteroids are to reduce vasodilation and capillary permeability, as well as to reduce the migration of leukocytes to inflammation [52]. Corticosteroids that bind to the glucocorticoid receptor mediate changes in gene expression that lead to multiple subsequent effects over several hours or days [52]. Corticosteroids inhibit apoptosis and demargination of neutrophils, and inhibit phospholipase A2, which reduces the formation of arachidonic acid derivatives [52]. Lower doses of corticosteroids have an anti-inflammatory effect, while higher doses have an immunosuppressive effect [52]. In addition, corticosteroids play a role in the regulation of cytokine balance in patients with IDD. They inhibit NF-κB and other inflammatory transcription factors, and corticosteroids also contribute to the modulating effects of anti-inflammatory cytokines (e.g., IL-10) [53]. Corticosteroids suppress the production of pro-inflammatory cytokines such as IL-1 and TNF-α by macrophages and monocytes through several mechanisms. One mechanism is the enhanced synthesis of the IκBα protein. IκBα forms inactive cytoplasmic complexes with NF-κB, which itself activates many immunoregulatory genes in response to pro-inflammatory cytokines [54]. Other mechanisms of action that have recently been reported are the down-modulation of histone acetyltransferase and up-regulation of histone deacetyltransferase, both of which negatively affect messenger RNA transcription [55], which may play an important role in the development and progression of IDD.

Prednisolone ((11beta)-11,17,21-trihydroxypregna-1,4-diene-3,20-dione) is a corticoid similar to cortisol [56]. Prednisolone has a complex anti-inflammatory effect, affecting both molecular and cellular components of the immune system [57]. It is involved in the regulation of cytokine balance. Thus, prednisolone acts through genomic pathways, such as a transcription factor, and induces or represses gene transcription by direct binding to response DNA and/or binding to other transcription factors [58]. A number of genes that are activated through the transactivation mechanism exhibit anti-inflammatory effects, but corticosteroid receptors can be regulated through transrepression and lead to an increase in the level of pro-inflammatory cytokines such as TNF-α, IL-12, and interferon gamma (IFN-γ) [59]. Transrepression may be mediated by a direct reaction of corticosteroid receptors with other transcription factors, including NF-κB, AP-1, CREB, NFAT, STAT-6, IRF-3, STAT-3, GATA-3, and T-bet [60]. Prednisolone inhibits NF-κB, suppressing the production of pro-inflammatory cytokines (IL-1, TNF-α, IL-6), and promotes an increase in the level of anti-inflammatory cytokines (IL-14, IL-10) [53]. Treatment with prednisolone and other corticosteroids resulted in selective survival of a specialized subpopulation of T cells, which play a key role in maintaining tolerance to autoantigens [61] and suppression of an excessive immune response [61], as well as increased production of immunosuppressive (anti-inflammatory) cytokines by degenerating IVD cells (IL-10, IL-35, TGF-β) [62].

Hydrocortisone ((11beta)-11,17,21-trihydroxypregn-4-ene-3,20-dione), or cortisol, is a corticoid secreted by the adrenal cortex. Hydrocortisone is used to treat immune, inflammatory, and neoplastic conditions [14]. Hydrocortisone plays a role in the regulation of cytokine balance in IDD. In an animal experiment, when using a dose of hydrocortisone, 5 μg/ml, there was a significant decrease in the concentration of pro-inflammatory cytokines (TNF-α, IL-1β, IL-1Rα, CCL2/MCP-1, CXCL8/IL-8, IL-6, and IL- 12) after stimulation with lipopolysaccharide. Glucocorticoid receptors counteract the activity of transcription factors such as NF-κB [63]. Inflammatory signals typically activate MAPK. Hydrocortisone stimulates the production of the anti-inflammatory protein MAPK phosphatase 1. Thus, the effect of hydrocortisone on cytokine imbalance in patients with IDD may be mediated through several mechanisms of inflammation control [64]. Hydrocortisone can influence apoptosis of IVD cells [65]. Its additive effect on the concentration of the pro-inflammatory cytokine TNF-α was found with a combination of low doses of infliximab and hydrocortisone, which may indicate a more pronounced immunosuppression [66].

#### 3.2.2. Melatonin

Melatonin (N-[2-(5-methoxy-1H-indol-3-yl)ethyl]acetamide) is an endogenous hormone produced by the pineal gland that regulates sleep-wake cycles and, when administered exogenously, has a beneficial effect on sleep onset latency. Melatonin is a derivative of tryptophan. It binds to the melatonin type 1A receptor, which then acts on adenylate cyclase and inhibits the cyclic adenosine monophosphate (cAMP) signal transduction pathway. Melatonin not only inhibits adenylate cyclase, but also activates phosphilpase C. This potentiates the release of arachidonic acid. By binding to melatonin receptors 1 and 2, the underlying signaling cascades have various effects on the body [14].

It has been shown that melatonin also plays a role in the regulation of cytokine balance. Thus, it is widely involved in the regulation of pro-inflammatory and anti-inflammatory reactions in various pathophysiological conditions, especially in the inhibition of the activation of the NLRP3-IL-1 axis [67]. Zhang et al. [68] found that melatonin administration can significantly inhibit the expression of pro-inflammatory cytokines IL-1β, IL-6, and TNF-α in IVD punctured rat tails. Melatonin protects the structural integrity of the IVD and inhibits the aggregation of inflammatory cells and the release of pro-inflammatory cytokines. Chen et al. [69] showed that by suppressing NF-κB signaling and mtROS production, melatonin significantly attenuated the activation of NLRP-3 and pro-inflammatory cytokines (IL-1β) in NP cells and blocked the IL-1β-NLRP-3 positive feedback loop. Meanwhile, melatonin administration suppressed the expression of NLRP-3, p20, and IL-1β in IVDs of IDD model rats. Melatonin inhibited the IL-1/NF-κB pathway and reduced the amount of mitochondrial ROS (mtROS), further slowing down the development of IDD by inhibiting the activation of the NLRP-3 inflammatory system [70].

Nicotinamide phosphoribosyltransferase (NAMPT) is a powerful activator of NLRP-3 [71]. Huang et al. [72] showed that melatonin can reduce NLRP-3 signaling by inhibiting NAMPT expression induced by the pro-inflammatory cytokine TNF-α in NP cells and block the activation of gasdermin D and caspase-1, thereby attenuating the inflammation-induced catabolism of extracellular matrix in IVD.

Taken together, melatonin may interfere with the production of pro-inflammatory cytokines, including IL-1β, IL-6, and TNF-α, and protect against inflammation-mediated IDD. Research by Huang et al. [72] suggested that melatonin markedly inhibited TNF-α-induced degradation of collagen II and aggrecan in NP cells. TGF-β has been reported to play a positive role in slowing down the development of IDD by promoting extracellular matrix regeneration and restoration of homeostasis in degenerating IVDs [73]. Turgut et al. [74] reported that after subcutaneous injection of melatonin (30 mg/kg/day), TGF-β expression in rat IVDs significantly increased, and histopathological changes in experimental IDD also markedly decreased. After that, Zhang et al. [68] demonstrated that intraperitoneal injection of melatonin (1 mg/kg/day) clearly protected the structural integrity of IVDs components in a rat tail puncture model and, in particular, inhibited local expression of the anti-inflammatory cytokine IL-6.

#### 3.2.3. Estrogens

Estrogens (a group of derivatives of cyclopentanooctohydrophenanthrene having an aromatic ring) regulate many physiological processes, including normal cell growth and development, as well as the regulation of tissue-specific genes in the reproductive organs, brain, immune system, and in the cardiovascular and skeletal systems [75]. In addition, estrogens modulate the onset and progression of a number of connective tissue diseases, including rheumatoid arthritis and IDD [76]. In vitro studies have shown that the direct chondroprotective role of estrogen may be due in part to the synthesis of glycosaminoglycans, which are an important component of IVD [77]. Estrogen also inhibits COX-2 mRNA expression in bovine articular chondrocytes as well as other tissues. This fact was associated with protection against ROS-induced damage to chondrocytes [78].

Estrogen levels in postmenopausal women are thought to correlate with the risk of developing and severity of IDD. Additionally, estrogen can regulate IVD content and metabolism [79]. Estrogen replacement therapy is used to treat symptoms associated with menopause and interesting clinical evidence suggests that estrogen has benefits for IVD [80], despite the fact that estrogen plays an important role in the treatment of various diseases such as breast cancer, osteoporosis in menopausal women, and gynecological diseases [81]. The protective effect of estrogen on IVD in vitro has been determined mainly using several cellular models, such as the inflammation model [82], the high glucose model [83], the starvation model [84], and the drug model [85].

Estrogens are involved in the regulation of cytokine balance. In vitro experiments performed in chondrocytes suggested that not only estrogens, but also raloxifene (a selective estrogen receptor inhibitor) may have a potential chondroprotective role in osteoarthritis and IDD [86]. When cells were co-incubated with raloxifene, a significant and dose-dependent increase in proteoglycans and a decrease in MMP-3 and NO, induced by the pro-inflammatory cytokine IL-1, were observed. Raloxifene significantly reduced the expression of the *NOS2* gene encoding iNOS, indicating a protective effect of the selective estrogen receptor modulator on cartilage [86]. On the contrary, some authors postulate that estrogen may also have a detrimental effect on chondrocyte metabolism [87]. This statement is based on data obtained from young rabbits that received either systemic estrogen replacement therapy [88] or intra-articular estrogens [87]. Unexpectedly, these animals showed damage to the cartilage tissue of the joints. At the cellular level, high doses of estrogen have been shown to affect IL-1-stimulated proteoglycan degradation and MMP production [89]. For humans, the available data are also conflicting. Thus, polymorphisms of the human *ER* gene and radiographic osteoarthritis of the knee joint have been studied in different populations with paradoxical results [90]. However, observational studies have shown a beneficial effect of estrogen replacement therapy in some types of osteoarthritis, and that estrogen supplementation reduces the severity of osteoarthritis [91]. A recent study showed that estrogen induces temporomandibular joint inflammation in a dose-dependent manner through the NF-κB pathway [92]. All of these observations indicate that both concentration and level of estrogen, as well as cartilage location, must be considered in order to understand the dual effect of estrogen on chondrocyte homeostasis. In addition, the addition of progestogens to estrogen during hormone replacement therapy may counteract the beneficial effects on cartilage of estrogens alone [93].

Estrogen may reduce apoptosis of IDD cells in several ways, including the inhibition of pro-inflammatory cytokines (IL-1β and TNF-α), reduction of catabolism due to inhibition of MMPs, activation of α2β1 integrin and IVD anabolism, activation of the PI3K/Akt pathway, reduction of oxidative damage, and stimulation of autophagy [94]. Estrogen can regulate extracellular matrix synthesis in the IVD through ERβ-p38 MAPK signaling [95] and facilitate apoptosis via ER-β to modulate oxidative stress levels [83]. Liu et al. [82] reported that estrogen can attenuate human NP cell apoptosis induced by the pro-inflammatory cytokine TNF-α in vitro. In another study, Wang et al. [96] showed that 17β-estradiol protected human NP cells from TNF-α-induced apoptosis by stimulating the PI3K/AKT pathway. In addition, Li et al. [97] found that the anti-inflammatory cytokine TNF-α stimulates the expression of p53 and p16 by NP cells of degenerating IVDs in rats, reduces the content of extracellular matrix (aggrecan and type II collagen), and generated ROS in experimental IDD. The estrogenic supplement enhances NP cell proliferation activity, stimulates the synthesis of aggrecan and collagen II, and inhibits the formation of ROS and phosphorus in the NF-κB/p65 pathways compared to a group of experimental animals with IDD treated with TNF-α. In addition, ICI 182780 (a kind of ERs antagonist) can block the protective effect of estrogen on TNF-α treated IVD cells.

Yang et al. [98] used the pro-inflammatory cytokine IL-1β to determine the effect of estrogens on NP cells in vitro. IL-1β stimulated high expression of MMP-3 and MMP-13 and suppressed the expression of tissue inhibitors of MMPs (TIMPs). The balance between MMPs and TIMPs that is disrupted by IL-1β may be a potential mechanism for the progression of IDD. Estrogen has also been shown to have a protective effect against NP cell apoptosis by regulating the balance between MMPs and TIMPs, as well as reversing the degradation of collagen II and agrecan biosynthesis in IVD [98]. Estrogen protects NP cells from apoptosis by activating autophagy, which plays a key role in cell survival under conditions of stress and starvation. In addition, fasting increases the expression of MMP-3 and MMP-13 in NP cells, and estrogen treatment can reverse the abnormal expression of these MMPs. Wang et al. [99] found that estrogen can reduce AF cell apoptosis in an experimental model of rat IDD induced by the pro-inflammatory cytokine IL-1β in a dose-dependent manner. In addition, Zhao et al. [100] found that estrogen can increase the ability of rat AF cells to attach to type I collagen by upregulating integrin α1.

### 3.3. Growth Factors

Numerous growth factors, including bone morphogenetic proteins, insulin-like growth factor-1 (IGF-1), and TGF-β, and others have been reported to have therapeutic effects that slow or reverse IDD [101].

#### 3.3.1. Bone Morphogenetic Proteins

Bone morphogenetic proteins are a subclass of TGF-β proteins [102]. Thus, they play an important role in the formation and maintenance of bones, cartilage, and muscles [103]. Bone morphogenetic proteins manifest their effects through binding to two types of transmembrane serine threonine kinase receptors known as BMPR-I and BMPR-II [102,103]. Once the receptors are activated, they phosphorylate Smad-1, Smad-5, and Smad-8 [102,103]. Phosphorylated Smad-1/5/8 complex with unphosphorylated Smad-4, and this complex moves from the cytoplasm to the nucleus, where it binds to various transcriptional coactivators (e.g., p300 histone acetyltransferase) and CBP [CREB-binding protein]) or corepressors (e.g., TOB [ERBB2 transducer] and SIP1 [SMN-interacting protein 1]) to regulate gene transcription [102,103].

Xie et al. [104] identified PUMA (p53 upregulated modulator of apoptosis)-dependent apoptosis signaling, which is initiated by a decrease in bone morphogenetic proteins in the pathogenesis of IDD. A decrease in bone morphogenetic proteins does not activate their receptors on the cell membrane, reducing Smad-1/5/8 phosphorylation and disrupting the assembly of the pSmad-1/5/8-HDAC1-Smad-4 transcription complex. Disruption of the pSmad-1/5/8-HDAC1-Smad-4 complex causes PUMA induction and PUMA accumulation, activating subsequent PUMA events, including the release of cytochrome C from mitochondria and activation of Apaf-1, caspase-9, and caspase-3, which eventually led to the development of IDD. PUMA is a critical mediator of apoptotic signaling and can be induced by various stimuli such as genotoxic stress, redox microenvironment, anti-inflammatory cytokine deficiency, and infection [105,106].

Bone morphogenetic proteins may be involved in the regulation of cytokine balance. A study by Tan et al. [107] demonstrated that bone morphogenetic protein 2 inhibits the expression levels of pro-inflammatory cytokines (TNF-α and IL-6) and increases the expression of the anti-inflammatory cytokine IL-10, which indicates that this protein can facilitate the degradation of extracellular matrix in IVD by regulating inflammatory factors. Considering these results, it is hypothesized that bone morphogenetic protein 2 may promote collagen and aggrecan synthesis and inhibit the degradation of extracellular matrix in IVD to inhibit the pathogenesis of IDD. Treatment with bone morphogenetic protein 2 significantly increased the levels of collagen type II, aggrecan, and SOX-9, but decreased the levels of MMP-13 and CTX-II in IDD rats and NP cells in a dose-dependent manner. At the same time, pretreatment with recombinant human bone morphogenetic protein 2 also significantly reduced the apoptosis rate of IL-1β-treated NP cells by reducing the level of cleaved caspase-3 and increasing the level of uncleaved poly(adenosine-5′-diphosphate-ribose) polymerase. It was demonstrated that recombinant bone morphogenetic protein 2 also significantly reduced the inflammatory response in NP tissues and cells, based on the levels of IL-6, TNF-α, and IL-10. In addition, recombinant bone morphogenetic protein 2 inhibited cell apoptosis by increasing phosphorylation levels of the PI3K/Akt signaling pathway, and pretreatment with LY29400 inhibited the effects of bone morphogenetic protein 2 in NP cells treated with IL-1β [107]. Although other studies using bone morphogenetic proteins such as bone morphogenetic protein 2 and bone morphogenetic protein 7 in various animal models of IDD have either shown minimal or no effect or reported severe ADRs [108,109].

#### 3.3.2. Transforming Growth Factor Beta

Transforming growth factor beta (TGF-β) is a multifunctional cytokine with three isoforms [110]. TGF-β proteins are produced by all lines of leukocytes. Activated TGF-β forms complexes with other factors to form a serine/threonine kinase complex that binds to TGF-β receptors. TGF-β receptors are part of subunits of type 1 and 2 receptors. After TGF-β binding, receptor kinase 2 phosphorylates and activates receptor kinase 1, which activates the signaling cascade. This causes the activation of various downstream substrates and regulatory proteins, inducing the transcription of various target genes, which are manifested in the differentiation, chemotaxis, proliferation, and activation of many immune cells [111].

TGF-β is involved in the regulation of cytokine balance. The canonical Smads signaling pathway is not the only signaling pathway regulated by TGF-β. Non-Smad signaling proteins can initiate parallel signaling that ultimately interact with Smads or interact with other major signaling pathways. Among them, the MAPK family, which includes extracellularly regulated kinases (ERK1 and 2), JNK, and p38 MAPK, play an important role in TGF-β signaling [112]. ERK types 1 and 2 are activated via the Raf-Ras-MEK1/2 pathway induced by mitogenic stimuli such as epidermal growth factor [113]. At the same time, JNK and p38 MAPK are activated by MAPK kinase, which are themselves activated by TGF-β-activated kinase-1 under stressful stimuli [114].

TGF-β1 plays a role in inducing the synthesis of pro-inflammatory cytokines by CD4^+^-induced T cells that perform a regulatory function, as well as by Th17 cells that secrete pro-inflammatory cytokines [115]. Only TGF-β1 accelerates the expression of FOXP-3 (forkhead box P3) and the differentiation of CD4+ T cells from activated T helper cells. TGF-β inhibits B cell proliferation. Additionally, TGF-β induces apoptosis of immature or resting B cells. TGF-β has been shown to suppress c-myc, as it does by inhibiting B cell proliferation.

It is known that TGF-β induces the NF-κB inhibitor IKBa, suppressing NF-κB activation [116]. NF-κB is a transcription factor that regulates the synthesis of pro-inflammatory cytokines (IL-1, TNF-α and defensins), although its function in apoptosis can be separated from this function. TGF-β stimulates resting monocytes and inhibits activated macrophages. For monocytes, TGF-β has been shown to act as a chemoattractant and also as an enhancer of the inflammatory response [117]. Additionally, TGF-β suppresses the production of pro-inflammatory cytokines in monocytes and macrophages, probably due to the aforementioned inhibition of NF-κB [118]. This controversy may be due to the fact that the effect of TGF-β is highly dependent on the context and clinical situation [119].

In the presence of IL-4 or anti-inflammatory cytokines (especially IL-6), TGF-β supports the formation of Th9 or Th17 cells, respectively [120]. Th cell functions are controlled by regulatory CD4+ T cells that express the FOXP-3 transcription factor [121]. Elucidation of the mechanisms that control CD4+ T cell homeostasis became even more important when it was found that CD4+ T cells that had lost FOXP3 expression could produce the pro-inflammatory cytokines IFN-γ and IL-17 [122]. On the one hand, the downregulation of FOXP3 may be necessary to attenuate the suppressive effect of CD4+ T cells, providing an effective immune response to pathogens. On the other hand, CD4+ T cell instability exacerbates tissue damage by IVDs and contributes to autoimmune pathology [123].

Thus, the regulation of Th cell line plasticity is of crucial importance for understanding the immune regulation and pathogenesis of autoimmune mechanisms of IDD development [124]. In cartilage, TGF-β has been shown to be involved in processes including cartilage formation, MMPs production, and inflammatory responses. TGFB gene expression has been found to be upregulated in experimental osteoarthritis cells similar to that observed in IDD. TGF-β and TGF-β type 2 receptors are commonly present in herniated IVDs cells and in non-herniated human IVD specimens [125]. Xie et al. [126] showed that TGF-β1 significantly reduced apoptosis in the NP of TNF-α treated rats and reduced the activity of caspase-3 and caspase-8. TGF-β1 attenuates autophagy and apoptosis induced by exogenous H_2_O_2_ by suppressing kinases regulated by extracellular signals in AF [127]. Single or repeated direct injections of TGF-β into the tissue of a degenerating human IVD in vitro contributed to an increase in proteoglycan synthesis and a decrease in tissue resorption due to a decrease in MMP-2 secretion, and also provided a transient proliferative effect [128].

#### 3.3.3. Insulin-like Growth Factors

Insulin-like growth factors (IGFs) are proteins with high sequence similarity to insulin. IGFs are part of a complex system called the IGF “axis” consisting of two cell surface receptors (IGF-1R and IGF-2R), two ligands (IGF-1 and IGF-2), a family of seven high-affinity IGF- binding proteins (from IGFBP1 to IGFBP7), as well as associated enzymes that destroy IGFBP [47].

The IGF “axis” is also commonly referred to as the growth hormone/IGF-1 axis. It is known that IGF-1 is important not only for the regulation of normal physiology, but also for the regulation of a number of pathological conditions. The IGF axis has been shown to play a role in promoting cell proliferation and inhibiting apoptosis. IGF-2 is considered to be the main growth factor required for early development, while the overexpression of IGF-1 is required to achieve maximum growth [129]. IGF-1 is involved in the regulation of neuronal development [130] and forms the development of the cochlea by controlling apoptosis [131].

The genetic and pharmacological suppression of the IGF-1 signaling pathway in numerous animal models has consistently shown that the downregulation of the IGF-1 signaling pathway leads to healthier aging and increased lifespan [132]. It has been suggested that a decrease in IGF-1 signaling reduces mTOR (mechanistic target of rapamycin) and switches cellular metabolism from cell growth to cell maintenance and repair activities, reducing the accumulation of senescent cells [133].

IGF-1 is a promising drug for the treatment of IDD because it is able to stimulate the anabolic production of extracellular matrix in IVDs [134]. Studies of IVD cell culture and IGF-1 stimulation showed increased proteoglycan synthesis by NP cells [135]. It has been shown that IGF-1 stimulates both DNA synthesis and downstream signaling in NP and AF cells in vitro [136]. There is evidence to support the use of IGF-1 for the treatment of patients with IDD in vivo based on reports of decreased serum IGF-1 levels in patients with IDD. These studies demonstrate an association of low circulating IGF-1 levels with an increased rate of IDD development [137].

In addition, a beneficial effect of IGF-1 on the reduction of senescent cells in human AF cells exposed to H_2_O_2_ has been shown, suggesting a protective effect of IGF-1 against oxidative stress observed in aging and IDD [138]. However, circulating IGF-1 levels are also affected by age, sex, and body mass index, all of which affect the risk of developing IDD [139,140]. It is likely that IGF-1 regulates aggrecanolysis in the IVD through its effect on cellular senescence, i.e., reduced IGF-1 signaling leads to a decrease in aggrecanolysis in the IVD by suppressing the development of early IVD cellular senescence. Senescent IVD cells have been shown to be catabolic, producing copious amounts of MMPs that enhance aggrecanolysis in IVD tissue [141]. It should be recognized that some previous studies have had conflicting results. Thus, studies using IMR-90 primary human fibroblasts and mouse embryonic fibroblasts have shown that long-term treatment with IGF-1 causes premature cell aging in a p53-dependent manner [142]. In contrast, Gruber et al. [138] reported that the addition of IGF-1 to IVD cell culture rescued AF cells from premature aging caused by oxidative stress.

Previous work has demonstrated the role of IGF-1 as an anabolic agent for IVD, able to stimulate both prostaglandin synthesis and IVD cell proliferation [134,135,136]. At the same time, IGF-1 stimulates the production of extracellular matrix in chondrocytes. Loeser et al. [143] reported a significant increase in the synthesis of prostaglandins by chondrocytes obtained from the knee joints of patients with osteoarthritis, which were cultured in alginate granules with exogenous IGF-1 (100–1000 ng/mL). Additionally, increased prostaglandin synthesis has been demonstrated in monolayer articular chondrocytes and explant models stimulated with IGF-1 [144]. Kritschil et al. [145] suggested that IGF-1 signaling is also required for in vivo IVD proteoglycon production, but long-term IGF-1 signaling increases the risk of cellular senescence and aggrecanolysis. Moreover, in order to synthesize new extracellular matrix in IVDs after IGF-1 stimulation, the cells of a degenerated or aged IVD must have the nutrients necessary to generate energy to perform these functions. However, IVD nutrient supply is reduced in most old and degenerated IVDs due to end plate calcification [146].

Several studies have shown that IGF-1 can promote extracellular matrix synthesis in IVD [147,148]. It also prevents the degradation of the extracellular matrix by inhibiting MMPs, which increases the amount of extracellular matrix in IVD and delays the progression of IDD. MMP-13 is known to be an important enzyme in the degradation of extracellular matrix components in IVD, such as collagen and proteoglycans [149]. In animals with *IGFIR* gene knockout, the amount of type II collagen and aggrecan gradually decreased, but MMP-13 mRNA expression increased depending on time [150]. Additionally, IGF-1 treatment significantly reduced MMP-3 expression levels and increased extracellular matrix levels in degenerating IVD animal models by knocking out the *LEPR* gene encoding the leptin receptor [151]. In addition, in vitro studies have shown that IGF-1 increases the synthesis of proteoglycans and inhibits the production of MMP-2 [152]. IGF-1 can not only inhibit catabolism, but also promote anabolism [151]. IGF-1 induces NP mesenchymal stem cells to synthesize the extracellular matrix by upregulating the expression of the chondrogenic genes *COL1A2, ACAN*, and *SOX9* via the ERK/MAPK signaling pathway [153].

A growing body of evidence indicates that IGF can slow down cellular apoptosis in IDD [148,151]. The introduction of IGF1 significantly reduced IL-1β-induced apoptosis of NP cells [154]. Type II diabetes mellitus induced by *LEPR* gene knockout in mice led to the development of IDD by promoting apoptosis of IVDs cells. Treatment with IGF-1 partially reversed this situation [151]. NP cells treated with an adenoviral vector expressing hIGF-1 showed a decrease in the rate of apoptosis of IVDs cells, confirmed by the TUNEL test (terminal deox-ynucleotided transferase-mediated dUTP-biotin Nick-End Labeling) and FCM (fuzzy classifier means) performed using TNF-α [155]. In addition, IGF-1 can also inhibit H_2_O_2_-induced cellular senescence [138].

Le Maitre et al. [156] found IGFR-1 expression in ingrowing blood vessels, which is part of the etiology of IDD. Thus, the addition of IGF-1 may exacerbate ingrowing angiogenesis in IDD. Zhu et al. [157] found higher levels of IGF-1 in NP cells in patients with a herniated IVD of the lumbar spine compared with controls, as well as a positive relationship between hernia severity and IGF-1 levels, indicating high IGF-1 ADRs. with IDD.

Studies have shown that IGF-1 can reverse the inhibition of IVD cell proliferation [144]. Mavrogonatou et al. [158] also reported that IGF-1 stimulated DNA synthesis in NP cells under various osmotic conditions via the ERK and Akt signaling pathways. These data indicate that IGF-1 plays a positive regulatory role in promoting cell proliferation. However, the hyperproliferation of IVD cells leads to an increase in food intake and demand, which is associated with the development of IDD [159].

On the other hand, the excessive activation of IGF-1 signaling may contribute to the development of IDD. In the case of pathological nutritional deficiencies, exogenous IGF-1 injection only benefits well-nourished IVD areas, while increasing IVD cell apoptosis in malnourished areas [160]. IGF-1 promotes cell proliferation by increasing proteoglycan production and promoting cellular metabolism, thus increasing the nutritional requirement of IVDs [161].

On the other hand, the injection of IGF-1 into human IVDs can cause unnecessary ingrowth of blood vessels and accelerate the process of IDD [150]. The activation of IGF-1 can result in the increased expression of the pro-inflammatory cytokines IL-1 and IL-2 via the PI3K/Akt signaling pathway in lumbar herniated IVDs [162]. These results indicate that abnormal activation of IGF-1 signaling pathways may accelerate the IDD process in some cases [163].

The role of IGF-1 in the regulation of cytokine balance continues to be studied. The Akt signaling pathway, which functions after IGF2-IGF1R, is involved in various cellular responses such as survival, proliferation, and inflammatory reactions [164]. Western blotting revealed an increase in the ratio of phosphorylated Akt to total Akt and phosphorylated NF-κB to total NF-κB after IGF-2 stimulation. In addition, IGF-2 stimulation increased the expression of the iNOS enzyme, which is regulated by NF-κB [165]. NO production via iNOS plays an important role in osteoclastogenesis [166], suggesting that activation of iNOS expression after IGF-2 stimulation is associated with a mechanism that promotes osteoclastogenesis. Given that iNOS-mediated NO production also increases *IGF2* gene expression [166], IGF-2 and iNOS can regulate their own expression in a positive feedback loop. Stimulation of IGF-2 increased the expression of genes encoding pro-inflammatory cytokines in osteoclast and non-osteoclast fractions. Thus, the expression of *IL1B*, *TNFA*, and *IL6* genes is regulated by Akt-NF-κB, which suggests that IGF-2 stimulation increases the expression of these pro-inflammatory cytokines by activating the Akt-NF-κB pathway. In addition, the pro-inflammatory cytokines IL1B, TNF, and IL6 promote osteoclastogenesis [167], suggesting that IGF-2 induces osteoclastogenesis by increasing the expression of these cytokines. In addition, the activation of the RANKL gene in a fraction other than osteoclasts may also promote osteoclastogenesis in the presence of IGF-2 in patients with IDD. Finally, IGF-2 promotes osteoclastogenesis under hypoxic conditions by increasing the production of pro-inflammatory cytokines and iNOS expression by activating the Akt-NF-κB pathway [168].

#### 3.3.4. Osteogenic Protein 1

Osteogenic protein 1 (OP-1), also known as bone morphogenetic protein type 7) is downregulated in the tissue of degenerating IVDs [169]. Recently, there is increasing evidence that OP-1 is effective in stimulating the synthesis of extracellular matrix in IVD and slowing down the development of IDD in animal models [170].

This protein belongs to the family of transforming growth factors-β (TGF-β) [171]. OP-1 synthesis is suppressed during the development of IDD, which is important to remember because OP-1 can significantly promote the synthesis of proteoglycans and collagen in degenerating IVD cells [169]. In addition, the delivery of OP-1 to the central part of the IVD improves its regeneration, which is reflected in the restoration of the IVD height and the enhancement of the signal intensity from the IVD during magnetic resonance imaging of the spine (in T2 mode) [172].

In addition, OP-1 is involved in the regulation of cytokine balance. Thus, the addition of OP-1 partially attenuates the effects of the pro-inflammatory cytokine TNF-α. It is hypothesized that OP-1 can alleviate inflammation induced by the pro-inflammatory cytokine TNF-α and slow down the aging of NP cells to some extent. For example, the use of OP-1 partially suppresses the expression of aging markers (p16 and p53) of the p53-p21-pRB pathways and the p16-pRB pathway, which determine replicative senescence and stress-induced premature aging, in TNF-α treated NP cells. This supports the hypothesis that OP-1 can suppress the effects of the pro-inflammatory cytokine TNF-α and slow down the TNF-α-induced senescence of NP cells. In addition, OP-1 may have other protective effects in IDD in vitro and in vivo: increased biosynthesis of extracellular matrix macromolecules in NP, suppression of NP cell apoptosis, and inhibition of pain-related behavior [173]. Additionally, OP-1 suppresses ROS generation in NP cells treated with the pro-inflammatory cytokine TNF-α, suggesting that OP-1 may alleviate TNF-α-induced inflammation in NP cells through the regulation of the ROS-/NF- pathway κB [174].

#### 3.3.5. Human Recombinant Growth Factors/Differentiation

Growth/differentiation factors (GDFs) were first identified as components of bovine cartilage and are members of the bone morphogenetic protein family. Most members of bone morphogenetic proteins (bone morphogenetic proteins-2/4, bone morphogenetic protein-7 (or OP-1), and bone morphogenetic proteins-9/10) exhibit strong bone-inducing activity, and GDF5/6/7 induces the formation of cartilage and tendons, but not bone tissue [175].

As with other members of the bone morphogenetic protein family, GDF signaling occurs through heteromeric transmembrane complexes of serine-threonine kinase receptors containing both type I and type II receptor molecules [176]. GDF-5 and GDF-6 act through type I receptors (1A and 1B), as well as through type II receptors of bone morphogenetic protein 2, type IIA activin A receptor, and type IIB activin A receptor [177]. When bound to the GDF receptor, they can recruit and activate the Smad1/5/8 pathways. Some bone morphogenetic protein molecules are also capable of signaling through non-Smad kinase cascades. In particular, p38 MAPK, Erk1/2, and JNK1/2.

GDF-5, -6, and -7 play a critical role in the development of bones, limb joints, skull, and axial skeleton [178] and are expressed in developing cartilage, tendons, and ligaments [179]. These results suggest that GDF-5 and GDF-6 are required for the normal development and maintenance of homeostasis of IVDs in mice and for the proper production of extracellular matrix by IVDs. In humans, mutations in the *GDF5* gene lead to various chondrogenic dysplasias, while some polymorphisms in this gene are associated with osteoarthritis. This may have important implications for the development and progression of IDD, which is closely related to the aging process.

A study conducted with recombinant GDF-5 showed that the stimulation of gene expression led to the synthesis of extracellular matrix proteins, type II collagen, and aggrecan in IVDs. Additionally, a restorative effect of GDF-5 injection has been shown in rabbit IDD models [180]. However, when this growth factor alone is administered, the long-term persistence of this drug in IVDs was significantly lower [181]. However, a clinical study of intradiscal administration of recombinant GDF-5 to assess the safety and tolerability of 2 doses (single administration) for the treatment of early stage of lumbar IDD did not give encouraging results. This suggests that treatment with recombinant GDF-5 either had no therapeutic effect in IDD or was accompanied by the development of ADRs [182].

GDF-5 has been shown to be closely associated with the development of IDD. Thus, an association between IDD and the risk allele of the rs143383 polymorphism of the *GDF5* gene was identified [183]. In degenerated IVD, especially in NP and AF, a decrease in *GDF5* gene expression was found. In addition, the extracellular matrix in IVDs of GDF-5 deficient mice is abnormal, and treatment with recombinant GDF-5 in vitro can increase the expression of genes encoding type II collagen and aggrecan, which are important structural components of IVDs [184]. The administration of GDF-5 or recombinant GDF-5 can lead to an increase in the height of the damaged IVD, as shown in an animal model of IDD. At the same time, recombinant GDF-5 can suppress the expression of MMP-3 [185], which plays an important role in collagen degradation in IVD. Thus, the adenoviral transduction of GDF-5 is effective in stimulating the secretion of the extracellular matrix by NP cells [186]. In addition to stimulating the synthesis of collagen and proteoglycans, adenoviral transduction of GDF-5 can enhance the expression of genes encoding type II collagen and aggrecan. The same positive therapeutic effect was obtained after direct injection of GDF-5 into a degenerated IVD in the experiment. Researchers injected recombinant GDF-5 into IDD mice and found that IVD height was significantly restored and expression of genes encoding type II collagen and proteoglycans was significantly increased [187]. In addition, there was an increase in the content of glycosaminoglycans and DNA, and an improvement in the index of differentiation of the extracellular matrix in IVD (the ratio of type II collagen to type I collagen).

GDF-5 can upregulate growth factors that promote stromal cell angiogenesis and promote extracellular matrix synthesis in IVD. Additionally, GDF-5 can expand the AF-fibrochondrocyte population to NP. At the same time, this factor can activate the expression of genes encoding proteoglycans, promote the synthesis of proteoglycans [188], and inhibit IVD end plate calcification.

In preclinical (animal) models of IDD, treatment with GDF-5 and GDF-6 has shown promising results. Initial studies examined the delivery of GDF-5 and GDF-6 by intradiscal injection in the IDD model. For example, in mouse models, IVD recovery after static compression was improved with a single injection of GDF-5. After 4 weeks, there was a significant increase in the height of damaged IVDs and the number of cells in the NP and in the internal AF, with cells expressing both aggrecan and type II collagen. Similarly, in puncture models of IDD in mice [189] and rabbits [190], GDF-5 administration improved IVD height and the histological appearance of IVD tissue samples [191].

Human recombinant growth/differentiation factors-5/6/7 play an important role in the regulation of cytokine balance. In vitro GDF-6 increases extracellular matrix production in degenerating IVDs [192], while in vivo GDF-6 treatment prevents IDD in a sheep model [193]. It has been shown that GDF-5 directly inhibits the expression of MMP-13 and ADAMTS-4 in human chondrocytes [194] and also inhibits the expression of pro-inflammatory cytokines TNF-α, IL-1β, and prostaglandin E2 in mouse NP cells [195]. At the same time, IL-1β and TNF-α were found to significantly reduce GDF-5 expression in human AF cells in 3D culture [196]. In a mouse model of IDD, plasma levels of pro-inflammatory cytokines were reduced by the overexpression of human GDF-6 [197].

It was shown that the injection of GDF-6 into NP tissues reduced the expression of genes encoding the pro-inflammatory cytokines IL-6 and TNF-α, with a tendency to decrease the expression of IL-1β and pain-associated molecules VEGF (vascular endothelium growth factor), prostaglandin- endoperoxide synthase 2, and nerve growth factor. Moreover, the transplantation of NP treated with GDF-6 significantly reduced allodynia associated with transplantation. Additionally, there was a corresponding decrease in the number of pain-associated calcium-binding adapter molecule-1 molecules and nociceptive neuropeptide associated with the calcitonin gene in GDF-6-treated NP-associated dorsal root ganglia [198].

GDF-5 can inhibit the activation of the NF-κB signaling pathway, reduce the transcription of pro-inflammatory cytokine genes, and suppress the expression of pro-inflammatory cytokines. The expression levels of the cytokines TNF-α, IL-1β, proteoglycan E2, and NO concentrations were significantly suppressed by GDF-5 overexpression. In addition, GDF-5 overexpression reduced the lipopolysaccharide-induced upregulation of TNF-α, IL-1β, PGE2, iNOS, COX-2, collagen-II, aggrecan, IκBα, and p-p65 expression levels in NP cells. Taken together, these results demonstrated that GDF-5 attenuated lipopolysaccharide-induced IDD by inhibiting the production and release of pro-inflammatory factors [199].

Other related members of the bone morphogenetic protein family, such as bone morphogenetic protein 2, have been shown to act as chemotactic signals for mesenchymal progenitor cells [200], and GDF-6 acts as a chemoattractant for NP cells in vitro [192]. However, the administration of supraphysiological doses of growth factors may not be effective for the long-term treatment of IDD, due to the short half-life of these growth factors. At the same time, large doses raise concerns about the reduced safety of this drug due to the development of ADRs. In addition, repeated injection into an IVD can compromise its mechanical integrity and has been shown to induce an inflammatory response at the injection site [198].

### 3.4. Chondroprotectors

The possibility of influencing the etiological mechanisms of IDD according to the principle of replacement therapy appeared with the development of chondroprotectors containing glucosamine and chondroitin sulfate, belonging to the pharmacological group of correctors of bone and cartilage metabolism [201]. An important role in maintaining the necessary osmotic pressure (elasticity of the extracellular matrix and collagen fibers) is played by chondroitin sulfate, which is part of the articular cartilage and has a chondroprotective, chondrostimulating pharmacological effect and stimulates regeneration. Medicines containing chondoitin sulfate act mainly on the cellular component of inflammation and stimulate the synthesis of hyaluronic acid and proteoglycans [202].

#### 3.4.1. Chondroitin Sulfate

Chondroitin sulfate (chondroitin-4-(hydrogen sulfate) is a chondroprotector with antiresorptive activity [203], anti-inflammatory and anti-aging effects [204], and symptom-modifying effects [205]. In addition to a direct effect on the severity of pain in IDD, chondroitin sulfate has a modulating effect on the level of systemic inflammation.

Chondroitin sulfate is involved in the regulation of cytokine balance. Thus, it plays a role in tissue remodeling, cell proliferation, migration and differentiation, suppression of apoptosis, and is also involved in the activation and deactivation of chemokines and proinflammatory cytokines by increasing the synthesis of hyaluronic acid and proteoglycans, suppressing the synthesis of prostaglandin E2, and cytokine expression, IL-1 and IL-6, as well as NF-κB, and COX-2 [206]. The antiresorptive-cytokine efficacy of chondroitin sulfate has been shown. Thus, against the background of combined therapy (intramuscular injections of chondroitin sulfate and NSAIDs), there was a decrease in the dose of NSAIDs and the serum level of pro-inflammatory cytokines 10 days after the start of therapy. Between days 10 and 60, there was a further decrease in the serum level of pro-inflammatory cytokines in the chondroitin sulfate group compared with the NSAID group [207]. Other studies have shown [208] the following effects of this chondroprotector: suppression of chondrocyte apoptosis by inhibiting NF-κB nuclear translocation in IL-1β-stimulated chondrocytes; stimulation of inhibition of prostaglandin, leukotrienes, and MMP metabolism proteins; inhibition of the effects of the transcription factor NF-κB and TNF-α; anti-inflammatory effect through inhibition of IL-1 induced expression of pro-inflammatory cytokines, chemokines, and growth factors; inhibition of angiogenesis in cartilage, increased synthesis of antiangiogenesis factors, and, thereby, reduction of inflammation; and reduction in the area of subchondral bone resorption. It is the antiresorptive activity of chondroitin sulfate that plays a role in tissue remodeling, proliferation, migration, and differentiation of IVD cells, suppression of apoptosis, as well as in the activation and deactivation of chemokines and cytokines by increasing the synthesis of hyaluronic acid and proteoglycans, suppressing the synthesis of prostaglandin E2 and the expression of pro-inflammatory cytokines, L-1 and IL-6, and the inhibition of NF-κB expression, and COX-2. An in vivo study noted the process of inhibition of NF-κB under the influence of chondroitin sulfate, as well as a decrease in the level of inflammatory biomarkers, C-reactive protein, IL-6, and NOSs [209].

One of the pilot studies assessed the antiresorptive-cytokine efficacy of chondroprotective therapy with chondroitin sulfate for nonspecific pain in the lower back and osteoarthritis of the knee [210]. The study included 116 patients who received intramuscular injections of chondroitin sulfate in combination with NSAIDs, and 115 patients who received only NSAIDs. Each of the patients was prescribed one of the NSAIDs: meloxicam (15 mg), nimesulide (200 mg), celecoxib (200 mg), or etoricoxib (60 mg) for 10 days. During the study, the content of TGF-β1, IL-1β, and IL-6 was determined in the blood serum. During therapy with chondroitin sulfate, there was a significant decrease in the level of pro-inflammatory cytokines TGF-β1, IL-1β, and IL-6 [206]. Oral chondroitin sulfate was superior to placebo in the treatment of osteoarthritis. A meta-analysis [211] showed the following results: preparations based on pharmaceutical chondroitin sulfate have a more pronounced effect on the treatment of pain (−37%) and functional state (−18%) than preparations of chondroitin sulfate of a non-pharmacological class; the effect of chondroitin sulfate is positive at 3 and 12 months both in relation to pain and functional status; the simultaneous administration of NSAIDs orally does not significantly change the effects of chondroitin sulfate in relation to pain. In addition, a study by Melgar-Lesmes et al. [212] demonstrated that cultures of coronary endothelial cells and monocytes in the presence of chondroitin sulfate secrete fewer pro-inflammatory cytokines.

#### 3.4.2. Glucosamine Sulfate

Glucosamine sulfate (2-amino-2-deoxy-beta-D-glucopyranose) chondro-protector is a precursor of glycosaminoglycans. The use of glucosamine can help repair cartilage and slow down the development of IDD. Thus, glucosamine sulfate is able to inhibit the processes of O-acetylglucosamination, which explains its effect on the suppression of inflammation in the tissues of the joint in patients with type 2 diabetes mellitus, which may contribute to the prevention of complications of type 2 diabetes [213], including IDD.

Glucosamine sulfate may be involved in the regulation of cytokine balance. For example, some in vitro studies show that glucosamine reduces inflammation through the inhibition of IFN-γ [214] and NF-κB subunit 65 [214,215], reducing the severity of pain. The pathogenetic rationale for the use of glucosamine sulfate in osteoarthritis and IDD lies in the anti-inflammatory effect associated with the suppression of the aseptic inflammation cascade due to the activation of NF-κB, including the inhibition of the expression of MMP, IL-1β and IL-8, COX-2, and TNF-α. In addition, being a glycosaminoglycan, glucosamine sulfate inhibits free radicals that can cause the destruction of cartilage and collagen, inhibits the activity of enzymes that cause damage to cartilage tissue, participates in the synthesis of glycosaminoglycans, and increases the production of intraarticular fluid. In addition, glucosamine plays an important role in synthetic processes in IVD cells [216].

The antithrombotic effects of microcrystalline glucosamine sulfate have been established. Glucosamine sulfate, prescribed for the treatment of osteoarthritis and IDD, has independent antithrombotic activity, due, in part, to the inactivation of the NF-κB signaling pathways in platelets. Although the antithrombotic effects of glucosamine sulfate are, on average, 1.5–3 times weaker than those of NSAIDs, administration of glucosamine sulfate in combination with NSAIDs may enhance its antithrombotic effect [217].

However, evidence for safety and efficacy appears to be provided for prescription formulations of microcrystalline glucosamine sulfate, which cannot be extrapolated to generics, nutraceuticals, or OTC products [218]. Although, in general, the results of previous studies indicate a synergistic effect of glucosamine sulfate and NSAIDs in osteoarthritis and IDD [219]. Chemoreactome evaluations of the anti-inflammatory effects of glucosamine sulfate and NSAIDs on cells in culture with respect to various cytokines showed that glucosamine sulfate and NSAIDs exhibited comparable effects on the inhibition of IL-8 α and β receptors. At the same time, NSAIDs significantly enhance the effects of glucosamine sulfate in inhibiting the expression/secretion of IL-5 [219].

### 3.5. Traps of Interleukin-1

Two receptors for IL-1 have been identified and cloned as type I and type II (IL-1RI and IL-1RII). These receptors are usually co-expressed in different cell types [220]. IL-1 signaling activity seems to be mediated exclusively through IL-1RI, while IL-1RII does not have a signaling pathway but acts as a bait for IL-1 in myelomonocytic cells, inhibiting its activity and preventing IL-1 binding with IL-1 RI [221]. Colotta et al. [222] found that IL-4, IL-13 [223], and dexamethasone [224] are potent inducers of IL-1 by trapping the expression and release of IL-1RII in human myelomonocytes. Upregulation of the RII bait IL-1 by IL-4, IL-13, and dexamethasone is genetically determined and depends on the synthesis of these proteins. The ability of some cytokines and corticoids to induce the expression and release of a false target for IL-1 may contribute to the anti-inflammatory activity of these agents [224,225]. Treatment of polymorphonuclear leukocytes with chemoattractants leads to massive (up to 90%), fast (noticeable within 30 s), and protein synthesis-independent release from the surface of polymorphonuclear leukocytes in the soluble form of the IL-1 bait RII, followed by re-expression of the newly synthesized receptor.

Molecules representing various classes of chemotactic agents, including formyl-Met-Leu-Phe (FMLP), C5a, leukotriene B4, platelet activating factor, and IL-8, caused a rapid decrease in IL-1 binding capacity by human polymorphonuclear leukocytes, a cell type predominantly expressing IL-1 trap RII [221].

### 3.6. Interleukins Regulating Pro-Inflammatory Cytokines

Regulators of the activity and expression of anti-inflammatory cytokines under physiological conditions and in pathology (for example, in IDD [6]) are anti-inflammatory cytokines.

#### 3.6.1. Interleukin 4 and Interleukin 10

IL-4 is a T cell-derived pleiotropic cytokine that can exert both suppressive and stimulatory effects on various cell types [226]. IL-4 is a potent anti-inflammatory cytokine that acts by inhibiting the synthesis of pro-inflammatory cytokines (particularly IL-1, TNF-α, IL-6, IL-8, and IL-12) by macrophages and monocytes [227]. In addition, IL-4 stimulates the synthesis of other inhibitors of pro-inflammatory cytokines, such as the IL-1 receptor antagonist (IL-1Ra), antagonists of the soluble type II IL-1 receptor and TNF-α receptors [228]. IL-4 suppresses MMP production and stimulates TIMP-1 production in human mononuclear phagocytes and cartilage explants, indicating a protective effect of IL-4 on ECM degradation in IVD [229]. Moreover, IL-4 inhibits both osteoclast activity and survival, thereby blocking bone resorption in vitro. Of great importance is the fact that IL-4 could not be detected either in the synovial fluid or in the surrounding tissues [55].

IL-10 is a pleiotropic cytokine that can have inhibitory or stimulatory effects on various cell types. It has been identified as a factor produced by mouse Th2 cells that inhibits cytokine production by Th1 cells. IL-10 stimulates the proliferation and cytolytic activity of CD8+ T cells and activates NK cells [230]. IL-10 is also known to be a potent anti-inflammatory cytokine that inhibits the synthesis of pro-inflammatory cytokines such as IL-1, TNFa, IL-6, and IL-8 by macrophages and monocytes [231]. In addition, IL-10 inhibits MMP, stimulates the production of TIMP-1 in human mononuclear cells, which indicates a protective effect of IL-10 in relation to degradation of the IVD extracellular matrix [232]. 

The development of IDD may be under the particularly strict control of the anti-inflammatory cytokines IL-4 and IL-10. Thus, treatment with anti-IL-4/anti-IL-10 shortly before the onset of the disease in an animal model of experimental arthritis accelerated the manifestation of the disease [233]. Consistent with these findings, predominantly Th1 responses to type II collagen were found at the onset of this disease [234]. It has been argued [235] that exposure to IL-4 can induce immune abnormalities through the increased development of Th2-like primary CD4 effector cells.

Cartilage and bone erosion are prevented by IL-4 treatment. The combination with low doses of prednisone enhanced the anti-inflammatory activity of IL-4. This may be an attractive alternative to the use of high doses of prednisolone because it helps to minimize ADRs in patients with IDD. Studies of murine collagen arthritis [236] have shown that TNF-α is important at the onset of the disease, while IL-1 is the dominant cytokine not only at the onset, but also during the progression of the disease (arthritis and concomitant cartilage destruction). Moreover, the TNF-α receptor-deficient mice showed a slower onset of arthritis, but once the joint was affected, the arthritis progressed to full manifestation and cartilage destruction, further emphasizing that the use of TNF-α is important in early stages of the disease, but TNF-α is not the dominant cytokine in preventing the progression and destruction of cartilage in advanced stages of IDD [55].

IL-10 was marginally effective against synovial arthritis at a dose of 1–5 µg/day [233]. Although, in combination with doses of prednisolone as low as 0.05 mg/kg, the development of experimental synovial arthritis was halted and cartilage destruction was greatly retarded. The greatly improved efficacy of this combination was reflected in achieving the desired reduction in IL-1 levels and an increase in synovial fluid IL-10 levels. Accordingly, synovial IL-10 and IL-1ra mRNA levels were elevated, and IL-1β mRNA levels were reduced [232]. It has been demonstrated that the onset of experimental synovial arthritis is under strict control of the anti-inflammatory cytokines IL-4 and IL-10, since the blockade of both IL-4 and IL-10 by antibodies accelerates the onset of the disease [233]. However, treatment with low doses of IL-4 did not show a suppressive effect on disease activity and joint pathology. Interestingly, the combination of low doses of IL-4 and IL-10 had a stronger anti-inflammatory effect and resulted in protection against cartilage destruction [233,237]. Systemic treatment with high doses of IL-4 (3 µg/day) at the immunization stage delays the onset and also reduces the severity of the disease. However, when the administration of IL-4 was stopped, the expression and activity of the disease accelerated rapidly and were indistinguishable from those in the control group [238].

Systemic treatment with IL-4 in experimental streptococcal arthritis in rats resulted in the suppression of disease activity and a decrease in the chronic destructive process, which led to a decrease in lesions [239]. This was associated with increased levels of IL-1Ra, a natural inhibitor of IL-1, consistent with studies in humans receiving systemic IL-4 [240]. However, it is unlikely that the two-fold increase in serum IL-1Ra levels found after exposure to IL-4 is sufficient to suppress arthritis.

As previously stated, the blockade of IL-1 by the use of anti-IL-1 antibodies or very high doses of IL-1Ra completely suppressed experimental synovial arthritis and resulted in complete protection against articular pathology [236]. IL-4 has been reported to inhibit bone resorption by inhibiting osteoclast development and activity in vitro [241]. Systemic treatment with IL-4 markedly reduced bone erosion, which was investigated by radiographic analysis and histopathology. In addition, IL-4 suppresses the production of pro-inflammatory cytokines IL-1, IL-6, TNF-α, and prostaglandin E2 in several cell types that play a role in bone resorption, including in IDD. Interestingly, corticosteroids not only inhibit TNF-α and IL-1, but also reduce the production of IL-1Ra and regulatory (anti-inflammatory) cytokines such as IL-4 and IL-10 [242]. This suggests that the overall effect of inflammation of the joints of the spine may be impaired due to an imbalance of protective cytokines, in particular, an absolute or relative decrease in the level of anti-inflammatory cytokines, which inhibit the production of TNF-α/IL-1, and also induce powerful regulators of scavengers such as soluble receptors for TNF-α and IL-1, as well as IL-1Ra [243]. In addition, IL-4 strongly reduces iNOS expression, thereby counteracting the inhibitory effect of IL-1 on chondrocyte proteoglycan synthesis, which is mainly mediated by NO [244]. This demonstrates the synergistic effect of combination therapy with low doses of prednisolone and IL-4. However, low doses of IL-4 monotherapy did not suppress the clinical activity of the disease. Although, when combined with prednisolone, the progression of cytokine imbalance and inflammation in the IVD can be completely stopped [55].

#### 3.6.2. Interleukin 12

IL-12 is an anti-inflammatory cytokine secreted predominantly by macrophages and dendritic cells in response to bacterial cell wall components. IL-12 stimulates proliferation and also activates and increases the cytotoxicity of NK cells and T cells, promoting the differentiation of the latter into Th1 cells. IL-12 induces the secretion of IFN-γ and TNF-α and has a synergistic effect with IL-18 [245].

In addition, IL-12 may play a critical role in the development of IDD, since the blockade of endogenous IL-12 completely prevents the onset of experimental arthritis [234]. It has been demonstrated in animal models that experimental collagen arthritis can be controlled with recombinant IL-12. Thus, a daily dose of 100 ng of IL-12 can significantly suppress inflammation [238]. In vitro studies have demonstrated the ability of IL-12 to induce a significant increase in the level of the anti-inflammatory cytokine IL-10 in activated macrophages [246], apparently as a negative feedback control to improve/counteract the excessive Th1-activation of IL-12. A suppressive effect on IL-12 can be achieved by using anti-IL-10 [238]. A significant increase in the level of corticosterone (from 244 nmol/L to 647 nmol/L) was induced by the introduction of IL-12 in an animal model. In line with the available data, it is tempting to suggest that the observed IL-12-mediated suppression of experimental collagen arthritis was due to IL-10/steroid synergism [232], which allows a new look at the possibility of using such a therapeutic strategy in IDD.

### 3.7. Monoclonal Antibodies

#### 3.7.1. Antibodies against Tumor Necrosis Factor Alpha

Antibodies to TNF-α affect the cytokine imbalance. Thus, anti-TNF-α treatment showed efficacy shortly after the onset of the disease but had little effect on fully developed experimental arthritis. Anti-TNF-α significantly reduces joint pathology, which was determined by a decrease in inflammatory cell infiltration and cartilage damage [247].

Infliximab (anti-(human tumor necrosis factor)) is a chimeric monoclonal IgG1 immunoglobulin and an antibody to TNF-α. At therapeutic concentrations, monoclonal antibodies induce apoptosis of monocytes in peripheral blood [248] and T cells [249]. It also reduces vascular cell adhesion molecule-1 (VCAM-1) and CD-40 expression on mucosal endothelium [250], also influencing wound healing by increasing TIMP-1 production, thereby reducing the activity of MMPs. Infliximab is involved in the regulation of cytokine balance. This monoclonal antibody has a significant effect against the symptoms of several chronic inflammatory diseases, including IDD and rheumatoid arthritis. It is also a potent inhibitor of both soluble and membrane bound TNF-α [251]. An additive effect on the concentration of TNF-α was found with a combination of low doses of infliximab and hydrocortisone, which may indicate a more pronounced immunosuppression. Using an in vitro model, Nesbitt et al. [66] demonstrated a significant decrease in IL-1β concentration in purified human monocytes when infliximab was added after lipopolysaccharide stimulation. However, in a study by Ruud et al. [252], IL-1β concentrations did not change significantly at all doses of infliximab. Vis et al. [253] analyzed bone mineral density in 36 patients with rheumatoid arthritis treated with infliximab for one year and reported the preservation of bone mass in the hip joints and increase in bone mass in the lumbar vertebrae.

Adalimumab (anti-(human tumor necrosis factor)) is an anti-TNF-α monoclonal antibody used to treat a wide range of inflammatory conditions. Adalimumab binds specifically to TNF-α and inhibits its interaction with TNF-α receptors on the cell surface, including the p55 and p75 receptors. Adalimumab also lyses cells expressing surface TNF in vitro in the presence of complement [254]. In addition, adalimumab alters the biological responses that are induced/regulated by TNF-α, including changes in the levels of adhesion molecules responsible for migration of leukocytes during inflammation (ELAM-1, VCAM-1 and ICAM-1 with IC50 1–2 × 10^−10^ M) [38]. Wijbrandts et al. [255] examined bone mineral density in 46 patients with rheumatoid arthritis treated with adalimumab. They compared bone mineral density of the femur at the time of initiation of adalimumab treatment with that measured 1 year after initiation and noted the possibility of preventing bone loss. Krieckaert et al. [256] reported that bone mineral density loss in the spine was reversed within 4 years of treatment with adalimumab, making this drug promising for the treatment of IDD and spinal osteoporosis.

Etanercept and certolizumab pegol (anti-(human tumor necrosis factor) anti-body) are anti-TNF-α monoclonal antibodies used to treat a wide range of inflammatory conditions. There are two different receptors for TNF: a 55 kDa protein (p55) and a 75 kDa protein (p75) [257]. Etanercept is a dimeric soluble form of the p75 TNF receptor that can bind to two TNF-α molecules, thereby effectively removing them from the circulation [258]. Certolizumab acts by binding to and neutralizing the soluble and membrane portions of TNF-α without inducing complement or antibody-dependent cytotoxicity due to the absence of an Fc region [259]. One of the additional features of certolizumab pegol is that, due to the presence of pegylation, it is distributed more significantly in inflamed tissues compared to other anti-TNF-α monoclonal antibodies, such as infliximab and adalimumab [259]. Gulyas et al. [260] studied changes in bone mineral density during long-term use (within one year) of etanercept or certolizumab pegol. The results showed that these drugs are effective not only in correcting cytokine imbalance in patients with arthritis, but also in maintaining bone mineral density in the lumbar vertebrae.

However, in a study by Orsolini et al. [261], who evaluated the short-term effects of anti-TNF-α monoclonal antibodies on bone mineral density in the lumbar vertebrae at 6 months in patients with rheumatoid arthritis, found opposite results. Patients treated with infliximab, adalimumab, etanercept, certolizumab pegol, and golimumab showed significant bone loss in the lumbar vertebrae. This decrease was associated with an increase in osteogenic and resorption markers, indicating that treatment with TNF-α inhibitors may lead to high bone turnover [262] during chronic therapy.

#### 3.7.2. Antibodies against Interleukin 1 Alpha and 1 Beta

Antibodies against IL1-α/β affect the cytokine balance. Treatment with anti-IL-1 α/β improved the severity of both early and advanced stages of arthritis. This apparent suppression of established arthritis was confirmed by the administration of high doses of IL-1Ra. Dose-response experiments have shown that optimal reduction of this pro-inflammatory cytokine requires continuous administration of the drug at a dose of 1 mg/day. Significant suppression of inflammation in the joints is observed with anti-IL-1β, although the elimination of the negative effects of overexpression of IL-1α and IL-1β still provides better protection. A different degree of TNF-α/IL-1 dependence was shown at different stages of arthritis. However, blocking TNF-α does not necessarily eliminate the negative effects of the pro-inflammatory cytokine IL-1. In addition, continuous high doses of IL-1Ra are required to block inflammation [247]. However, these results explain the promise of using anti-IL-1 α/β to correct severe cytokine imbalances in severe IDD.

Canakinumab is a monoclonal antibody to human IL-1β of the IgG1/κ isotype. This drug is used in inflammatory diseases associated with cryopyrin-associated periodic syndromes, familial Mediterranean fever, and systemic juvenile idiopathic arthritis [14]. The possibility of using canakinumab in IDD needs to be studied.

Anakinra is a recombinant form of human IL-1 receptor antagonist. This drug is used to treat rheumatoid arthritis, a multisystem inflammatory disease occurring in the neonatal period, and IL-1 receptor antagonist deficiency. Anakinra competes with IL-1 and inhibits it by binding to the IL-1 receptor [263]. The use of anakinra in IDD has not yet been studied.

Rilonacept is a dimeric fusion protein consisting of IL-1R portions and an IL-1R accessory protein associated with the Fc portion of immunoglobulin G1. This drug acts as an IL-1 inhibitor and is used to treat cryopyrin-associated periodic syndromes. Rilonacept blocks IL-1β signaling by acting as a soluble decoy receptor that binds IL-1β and prevents it from interacting with cell surface receptors. Rilonacept also binds IL-1α and an IL-1 receptor antagonist (IL-1ra) with reduced affinity. Use in severe IDD is being studied [14].

#### 3.7.3. Trap Receptors for Pro-Inflammatory Cytokines

Trap receptors and regulators-sIL-1R2 and IL-1R8-affect the regulation of cytokine balance. High levels of sIL-1R2 attenuate IL-1β-mediated inflammation and antimicrobial response, including the production of pro-inflammatory cytokines IFN-γ and TNF-α [264]. Thus, an increased susceptibility to arthritis in mice with IL1r2−/− knockout was confirmed, which was associated with the increased production of inflammatory mediators, such as IL-6, CXCL2, iNOS, and IL-1β, by IL-1R2-deficient macrophages [265]. In addition, neutrophils act in trans to release sIL-1R2, which in turn inhibits IL-1β-mediated fibroblast activation, thereby regulating the expression of inflammatory molecules (e.g., IL-1β, IL-6, CXCL- 1, and CXCL). In contrast, IL-1R2 deficiency does not affect the severity and mortality after a single administration of IL-1β, suggesting that IL-1R2 is primarily involved in the regulation of local inflammation [266].

Polymorphonuclear-induced vascular permeability was reduced in transgenic mice infected with IL-1R2 [267]. Based on the high expression of the IL-37//IL-1R5//IL-1R8 complex in CD4+ cells of patients with rheumatoid arthritis, IL-37 has been proposed as a promising therapeutic target in rheumatoid arthritis [268]. In psoriatic arthritis, the peripheral blood mononuclear cell gene expression profile in patients and healthy individuals has shown that negative regulators of innate responses, including IL-1R8, are controlled by genes whose expression is most downregulated [269]. Consistent with these data, IL-1R8-deficient mice developed severe psoriatic joint inflammation in both chemical and cytokine-induced murine experimental psoriasis models, compared to wild-type mice [270]. In addition, IL-1R8 negatively regulates IL-36-dependent psoriatic inflammation in humans and mice, acting in particular on dendritic cells and keratinocytes and modulating neutrophil chemoattractants [271].

In general, IL-1R8 and IL-1R2 act as tuners in various physiological and pathological conditions, performing important functions in preventing immunopathology, including cytokine imbalance in IDD. On the other hand, their regulatory role may be exploited by escape mechanisms developed by pathogens, suggesting that their cell- and context-specific functions should be analyzed in the future to develop innovative IDD therapeutic strategies [272].

#### 3.7.4. Antibodies against Interleukin 6

Anti-IL6 antibodies affect cytokine balance. In *IL6* knockout mice, osteoclast apoptosis is stimulated and bone mass increases [273]. In addition, studies of antibodies to the IL-6 receptor administered to an animal model of arthritis have shown increased bone mineral density, suppression of bone loss in the lumbar vertebrae, and reduced markers of bone resorption [274].

Tocilizumab is an IL-6 receptor antagonist used to treat cytokine release syndrome, systemic juvenile idiopathic arthritis, giant cell arteritis, and rheumatoid arthritis [14]. Abu Shakra et al. [275] examined the rate of change in bone mineral density from baseline at 96 weeks in patients with rheumatoid arthritis treated with tocilizumab. The authors showed that the bone mineral density in the lumbar vertebrae was preserved. Additionally, Kume et al. [276] analyzed bone mineral density in the lumbar vertebrae at 52 weeks in patients treated with tocilizumab. Bone mineral density increased in patients whose bone mass was initially reduced. However, Briot et al. [277] found no change in bone mineral density in the lumbar vertebrae in patients with rheumatoid arthritis treated with tocilizumab for 48 weeks.

Sarilumab is an anti-IL-6R IgG1 monoclonal antibody that binds to both membrane-bound and soluble forms of the IL-6 receptor. This, in turn, leads to the blockade of cis- and trans-inflammatory signaling cascades of IL-6 [278]. Several clinical studies have shown that sarilumab treatment reduces serum levels of RANKL (receptor activator of nuclear factor kappa-B ligand) [279,280]. One of these studies, similar to tocilizumab, analyzed levels of osteogenic markers and found them to be elevated compared to controls [280].

#### 3.7.5. Janus Kinase Inhibitors

JAK inhibitors are small molecule compounds that inhibit the JAK family (a class of non-receptor tyrosine kinases that mediate signaling of inflammatory cytokines such as interleukins and interferons) [262].

Tofacitinib is an inhibitor of JAK 1, 2, and 3 types. Tofacitinib is a partial and reversible JAK inhibitor that prevents the body from responding to pro-inflammatory cytokine signals. By inhibiting JAK, tofacitinib prevents STAT phosphorylation and activation by inhibiting the JAK-STAT pathway and reducing the inflammatory response [14]. Tofacitinib has been reported to suppress osteoarticular destruction in a rat model of adjuvant arthritis [281]. However, in vitro studies have shown that tofacitinib does not affect the differentiation and function of human osteoclasts induced by M-CSF and RANKL but inhibits T-lymphocyte production of RANKL [282]. In contrast, tofacitinib has been reported to interfere with human osteoclast-like cell differentiation induced by M-CSF, TNF-α, and IL-6 [283]. Tofacitinib has also been shown to promote osteogenesis by stimulating the differentiation of mesenchymal stem cells into osteoblasts [284] and activation of the canonical Wnt pathway in osteoblasts [285].

Baricitinib is a JAK type 1 and 2 inhibitor. By inhibiting the action of JAK1 and JAK-2, baricitinib attenuates JAK-mediated inflammation and immune responses [38]. Baricitinib has been shown to suppress osteoarticular destruction in a rat and mouse model of arthritis [286]. Baricitinib has also been reported to indirectly inhibit osteoclast differentiation by downregulating RANKL expression on osteoblasts in a mouse osteoclast coculture system. However, baricitinib does not directly inhibit osteoclast differentiation [287]. In addition, baricitinib has been shown to promote osteogenesis by activating the canonical Wnt pathway in osteoblasts [285].

Peficitinib is an inhibitor of JAK types 1, 2, and 3 and tyrosine kinase 2, thereby attenuating cytokine-mediated inflammatory responses. Peficitinib has been reported to suppress osteoarticular destruction in a rat model of adjuvant arthritis [288]. However, the effect of peficitinib on vertebral bone metabolism has not been studied.

Filgotinib is the preferred JAK-1 inhibitor. Filgotinib acts on the JAK-STAT pathway by selectively inhibiting JAK-1 phosphorylation and preventing STAT activation, which ultimately leads to a decrease in pro-inflammatory cytokine signaling [289]. Filgotinib has been reported to suppress osteoarticular destruction in a mouse model of synovial arthritis. In addition, a decrease in RANKL gene expression in the extremities was demonstrated in the filgotinib group [290].

Upadacitinib is the preferred JAK-1 inhibitor. In cellular assays of human leukocytes, upadacitinib inhibited JAK-1/3-induced STAT-3/5 phosphorylation mediated by IL-6/7 [38]. A detailed analysis of the effect of upadacitinib on bone metabolism has not been performed.

Also of interest are the effects of the respective JAK inhibitors on Foxm1-positive osteoclasts and DC-OCs that occur in rheumatoid arthritis [262]. This group of drugs is promising for the correction of severe cytokine imbalance in patients with IDD, but so far, their effects in this disease have not been studied.

#### 3.7.6. Antibodies against Interleukin 17

IL-17 is a T cell cytokine produced by activated T cells, predominantly activated by CD4, CD45RO memory T cells [291]. In contrast to the limited expression of IL-17, the IL-17 receptor is ubiquitously expressed in almost all cells and tissues of the human body. It is a type I transmembrane protein with no sequence similarity to any other known cytokine receptor [292]. Binding of IL-17 to its unique receptor results in the activation of the adapter molecule TNF-α receptor-related factor 6, which is required for IL-17 signaling [293]. In addition, IL-17 can stimulate the production of pro-inflammatory cytokines IL-1 and TNF-α from macrophages [294]; it induces synoviocyte production of IL-6, IL-8, granulocyte-macrophage colony-stimulating factor, and prostaglandin E2 in humans [295]. This suggests that IL-17 may be a mediator of chronic inflammation as one of the mechanisms of IDD pathogenesis.

In vitro, IL-17 suppresses the synthesis of the extracellular matrix of articular chondrocytes by increasing the production of NO [296]. In addition, in vitro studies have demonstrated the role of IL-17 in bone erosion by the induction of the NF-κB receptor ligand activator (RANKL) [297]. IL-17 may promote joint inflammation as well as tissue destruction during the initial phase of arthritis. However, the role of IL-17 in the effector phase of arthritis has not yet been clarified.

Because a subset of patients which do not respond to IL-1 or TNF-α neutralization, other factors (e.g., IL-17) may be involved in the pro-inflammatory cytokine cascade. Thus, the participation of IL-17 in local inflammation in protracted arthritis has been shown. However, IL-17 has a dual effect on cartilage. In vitro-inhibits the metabolism of chondrocytes in intact mouse articular cartilage and leads to the breakdown of proteoglycans [298]. In vitro studies show that IL-17 induces MMPs in synoviocytes and chondrocytes [299]. In addition, the IL-1-independent role of IL-17 in the pathogenesis of arthritis in vivo has been shown [300]. IL-1 is a much more active catabolic cytokine in experimental arthritis than TNF-α, but IL-17 interacts with TNF-α to cause cartilage destruction in vitro [301]. This highlights the ability of IL-17 to act additively or even synergistically with IL-1/TNF-α, but IL-17 may have direct catabolic effects. A clear decrease in the expression of synovial IL-1 was found after the neutralization of endogenous IL-17, indicating that IL-17 is an upstream mediator of the pro-inflammatory cytokine IL-1 [302].

Antibodies against IL-17 are involved in the regulation of cytokine balance. Early neutralization of endogenous IL-17 prior to the development of inflammation in IDD in an experimental model of arthritis suppresses the onset of the disease [300,303]. In addition, a decrease in the number of multinucleated cells after neutralization of endogenous IL-17 was shown, indicating that treatment with antibodies against IL-17 prevents the formation of osteoclast-like cells. Interestingly, systemic treatment with osteoprotogerin inhibits local bone resorption induced by IL-17. It is suggested that IL-17-induced bone erosion is at least partially mediated by RANKL [304]. The observation that the neutralization of IL-17 results in a lower presence of RANKL-positive cells in the synovium suggests a link between IL-17 and RANKL expression. This highlights the role of IL-17 in enhancing the process of joint destruction and makes IL-17 an attractive target for the treatment of IDD. The neutralization of RANKL/RANK by the administration of osteoprotogerin prevents bone destruction [305]. However, such treatment is not anti-inflammatory or chondroprotective, as shown in the anti-IL-17 study, suggesting anti-IL-17 as a more appropriate therapy for IDD. For example, Lubberts et al. [302] used anti-IL-17 to neutralize endogenous IL-17 at the onset, after, and during the late stage of experimental arthritis. Anti-IL-17 injection appears to be much more effective than soluble IL-17 receptor (sIL-17R) treatment [303].

Presumably, the neutralization of IL-17 leads to a decrease in the expression of IL-1 in the synovium in the late stage of arthritis. However, IL-1 expression is not entirely dependent on IL-17. Blocking IL-17 with sIL-17R further improves the neutralizing effect of TNF-α blocking after the onset of arthritis. The mechanisms responsible for slowing disease progression after IL-17 neutralization in advanced arthritis may be applied to the suppression of other pro-inflammatory cytokines such as IL-1, TNF-α, and IL-6 [302], making anti-IL-17 a new a promising strategy for the treatment of cytokine imbalance in patients with IDD.

Secukinumab is a fully human anti-IL-17A IgG1/κ monoclonal antibody [38]. By blocking the action of IL-17A, secukinumab works to inhibit pro-inflammatory cytokines that cause immune-mediated inflammatory diseases [306]. Additionally, IL-17A is more potent than IL-17F, another member of IL-17, with much higher affinity for the IL-17 receptor [307].

Ixekizumab is a humanized anti-IL-17A IgG4 monoclonal antibody (mAb) that prevents its interaction with the IL-17A receptor. By blocking the action of IL-17A, ixekizumab works to inhibit pro-inflammatory cytokines that cause immune-mediated inflammatory diseases [14].

Brodalumab binds with high affinity to the IL-17A receptor, thereby inhibiting several pro-inflammatory cytokines from the IL-17 family [14].

### 3.8. Cytostatics

Methotrexate (N-[4-[[(2,4-diamino-6-pteridinyl)methyl]methylamino]benzoyl]-L-glutamic acid) is positioned as an anchor drug in antirheumatic therapy [308]. Methotrexate is a folic acid derivative that inhibits enzymes responsible for nucleotide synthesis, including dihydrofol reductase, thymidylate synthase, aminoimidazole-caboxamide ribonucleotide transferase, and amidophosphoribosyltransferase. Inhibition of nucleotide synthesis prevents cell division. This inhibition results in the accumulation of a ribonucleotide, aminoimidazole-caboxamide ribonucleotide transferase, which inhibits adenosine deaminase. In turn, this leads to the accumulation of adenosine triphosphate and adenosine in the extracellular space, stimulating adenosine receptors, which leads to the anti-inflammatory effect of this drug [309].

The effect of methotrexate on bone metabolism has been previously studied and demonstrated to prevent a decrease in osteogenesis and an increase in bone resorption, thereby maintaining bone mineral density in an animal model of arthritis [310]. Methotrexate directly inhibits osteoclastogenesis by decreasing the level of membrane TNF-α and (RANKL)-induced calcium influx into osteoclast progenitors [311]. Methotrexate has been shown to suppress the production of pro-inflammatory cytokines such as IL-1, TNF-α, IL-17, and IL-6, and to increase bone mineral density compared to untreated arthritis animal models [312]. In addition, animals treated with methotrexate showed a marked decrease in NF-κB, COX-2, and TNF-α translocation, accompanied by a significant decrease in NF-κB, TNF-α, and COX-2 in inflamed joints. These results are consistent with studies showing that methotrexate attenuates NF-κB, TNF-α, and COX-2 expression in rats with synovial arthritis [313] and partially regulates serum COX-2 activity in patients with rheumatoid arthritis [314]. Methotrexate can counteract joint inflammation in mice with synovial arthritis, as evidenced by the reversal of all elevated levels of pro-inflammatory cytokines (IL-1β, IL-6, IL-23, and IL-17) in inflamed paw tissue. Methotrexate can reduce plasma levels of TNF-α, IL-1β, IL-6, IL-17, and IL-23 in arthritis patients [315], in addition to serum levels of TNF-α and IL-6 in rats with synovial arthritis [316].

Cytostatics may be considered as possible drugs to correct severe cytokine imbalance in IDD, but their use is limited by possible ADRs.

### 3.9. Phosphodiesterase Inhibitors

3′,5′-cyclic nucleotide phosphodiesterases (PDEs) are highly conserved enzymes that catalyze the conversion of cyclic adenosine monophosphate (cAMP) and cyclic guanosine monophosphate (cGMP) to 5′-AMP and 5′-GMP, through which mammalian cells regulate intracellular levels of cAMP and cGMP. Therefore, cAMP is known to be an important second messenger that negatively regulates the synthesis and release of pro-inflammatory mediators and cytokines [317].

Several synthetic PDE-4 inhibitors have been identified that increase intracellular levels of cAMP, resulting in the decreased synthesis of pro-inflammatory cytokines (TNFα, IL-17 and IL-23). At the same time, PDE-4 inhibitors induce the synthesis of anti-inflammatory mediators, such as an antagonist of IL-10 and IL-1 receptors. In general, the anti-inflammatory effects of PDE-4 inhibitors are mediated by the modulation of Epac1/2 (activated cAMP ½), protein kinase A, and NF-κB-dependent signaling in various immune cells such as T-lymphocytes, monocytes, and dendritic cells. They have been shown to reduce inflammation associated with obesity by enhancing lipolysis, increasing brown adipose tissue formation, and reducing macrophage infiltration in white adipose tissue [318]. Taken together, the increase in cAMP levels due to PDE-4 inhibition is closely associated with the suppression of pro-inflammatory reactions. To date, two compounds with PDE-4 activity are apremilast and roflumilast.

Apremilast (N-{2-[(1S)-1-(3-ethoxy-4-methoxyphenyl)-2-(methanesulfonyl)ethyl]-1,3-dioxo-2,3-dihydro-1H-isoindol-4-yl}acetamide) is a selective PDE-4 inhibitor that blocks the synthesis of several pro-inflammatory cytokines and chemokines (TNF-α, IL-17, IL-12/IL-23p40, and CXCL8) in various cell types (e.g., T cells and macrophages) by increasing the intracellular level of cAMP [319]. Apremilast suppresses doxorubicin- and carfilzomib-induced myocardial inflammation in vivo by reducing metabolic stress by inhibiting caspase-3-dependent apoptosis and downregulating NF-κB, JNK, and p42/44 expression [320]. Consistent with these biological data, Mazzilli et al. [321] showed that apremilast positively regulates metabolic biomarkers of inflammation. Apremilast at medium doses (20–40 µM) has distinct anti-inflammatory properties without any cytotoxic effects, due in part to the suppression of GM-CSF, CCL2, CXCL10, VCAM-1, and E-selectin in TNF, through the differentiated inhibition of signaling NF-κB- and MAPK pathways, and decreased adhesion of Th-1 monocytic cells and effector memory T cells [322]. Apremilast reduces TNF-α-induced JNK, p38 phosphorylation and activation of NF-κB signaling, while p42/44 phosphorylation was not observed. GM-CSF expression is regulated through JNK-, p38-, and NF-κB-dependent pathways. The inhibition of p38 and NF-κB leads to the inhibition of TNFα-induced expression of the VCAM-1 protein, while the induced expression of E-selectin was blocked by treatment with specific inhibitors of JNK and p38. Treatment with high doses of apremilast (300 µM) has been shown to inhibit the expression of several pro-inflammatory cytokines (e.g., CXCL8) in prostaglandin E2-activated human epidermal keratinocytes without any cytotoxic effects and regardless of cAMP accumulation. In addition, it has been reported that various PDE-4 inhibitors (apremilast, roflumilast, and crisaborol) did not affect NF-κB signaling and that only apremilast abolished MEK-dependent pathways [323]. GM-CSF, VCAM-1, and E-selectin are differently regulated by MAPK and NF-κB signaling pathways in activated HUVECs (full name). Apremilast inhibits the secretion of pro-atherogenic IL-6 and CCL2 activated by IL-17A, while roflumilast was unable to suppress these factors, suggesting that the inhibition of these cytokines/chemokines occurs in part through a PDE-4 inhibitor-autonomous mechanism [322].

Tadalafil ((6R,12aR)-6-(1,3-benzodioxol-5-yl)-2-methyl-2,3,6,7,12,12a-hexahydro-2-methylpyrazino[1′,2′: 1,6]pyrido[3,4-b]indole-1,4-dione) is a phosphodiesterase-5 (PDE-5) inhibitor reported to exhibit anti-inflammatory activity in various experimental models including the treatment of autoimmune joint diseases [324]. Tadalafil regulates cytokine balance. Thus, in rats treated with tadalafil, there was a significant decrease in serum rheumatoid factor, pro-inflammatory cytokines IL-6 and TNF-α, MMP-3, and RANKL. At the same time, the level of anti-inflammatory cytokine IL-10 in serum increased. In addition, markers of inflammation and oxidative stress in the joint tissue (p-JAK2, p-STAT3, iNOS, NO, MPO, and MDA) decreased along with increased eNOS expression and glutathione content. The analgesic effect of tadalafil on zymosan-induced arthritis was associated with a decrease in neutrophil infiltration and a decrease in TNF-α levels [325]. Tadalafil partially protects against arthritis caused by collagen induction by improving the balance between oxidants and antioxidants [326]. The tadalafil-mediated decrease in MMP-3 levels may be associated with an increase in cGMP levels due to PDE-5 inhibition by tadalafil [327]. In addition, tadalafil activation of the PDE (phosphodiesterase 2) enzyme is one of the mechanisms by which RANKL stimulates monocyte chemotaxis and, subsequently, bone inflammation and bone destruction by osteoclasts [328]. Tadalafil has been shown to improve fractured bone healing by stimulating bone formation [329], which can be explained by the increased expression of eNOS, which enhances bone formation by activating osteoblasts [330]. These results are consistent with studies showing that cGMP can reduce TNF-α levels and hence NO released by neutrophils via iNOS in inflamed joints [331]. Thus, the regulation of NO synthesis and suppression of pro-inflammatory cytokines may be related to the role of cGMP, which accumulates during treatment with PDE-5 inhibitors such as tadalafil [316], the use of which in severe IDD needs to be studied.

### 3.10. Anticonvulsants and Antidepressants

Peripheral cytokine levels are also being studied in connection with the treatment of chronic pain in patients with severe IDD with anticonvulsants with a normothymic effect and antidepressants [332]. The relationship between acute and chronic inflammation is associated with depression, as the relationship between pro-inflammatory cytokines and depression has been studied for a long time [333]. It has been found that the levels of pro-inflammatory and anti-inflammatory cytokines change significantly with depression, and patients, even if they are otherwise clinically healthy, show elevated or decreased levels of various cytokines and their soluble receptors in body fluids [334]. There are stronger cytokine responses to pathogens and stressors in depression in patients with chronic discogenic back pain, in which, as a result of chronic cytokine imbalance, a pro-inflammatory response causes an anti-inflammatory response. These data complement the theory of two opposing counterregulatory systems: the immune-inflammatory response system and the compensatory immune-regulatory reflex system [335] in IDD patients with chronic back pain. In combination with other predisposing factors, these reactions lead to a persistent imbalance between pro-inflammatory and anti-inflammatory cytokines, the long-term dysregulation of various axes, stress, pain, mood changes, anxiety, and, ultimately, depression [336]. It is known that neuroinflammation is associated with the pathophysiology of disorders of the central nervous system, including depressive disorders in patients with IDD, which are independent risk factors for the development of cytokine imbalance, which in turn determines the clinical picture of IDD [337].

Carbamazepine (5H-dibenz[b,f]azepine-5-carboxamide is an anticonvulsant, normothymic, and analgesic [338]. Carbamazepine is chemically related to tricyclic antidepressants [339]. Its mechanism of action is to block voltage-dependent Na+ channels, which inhibits over-activation of neurons without interfering with normal neuronal signaling [340]. Carbamazepine has been shown to inhibit the lipopolysaccharide-activated expression of phospho-Act in BV-2 microglial cells [341]. Carbamazepine reduces the expression of TNF-α and IL -1β induced by lipopolysaccharide in the rat hippocampus [342]. Carbamazepine alleviates neuronal hyperinflammation by reducing levels of pro-inflammatory cytokines (IL-1β, IL-6, and TNF-α), especially when carbamazepine is combined with imipramine [343]. However, the effect of carbamazepine on cytokine status in patients with IDD is not well understood.

Imipramine (10,11-dihydro-N,N-dimethyl-5H-dibenz[b,f]azepine-5-propanamine) is a prototype of tricyclic antidepressants [344], structurally similar to phenothiazines [345]. Imipramine reduces the uptake of norepinephrine and serotonin by neurons by blocking Na^+^-dependent serotonin and norepinephrine transporters [346], which increases their concentration in the synaptic cleft and alleviates symptoms of depression [347,348]. In depressed individuals with chronic back pain, imipramine has a positive effect on mood because imipramine and amitriptyline are more potent serotonin reuptake inhibitors than other tricyclic antidepressants (e.g., nortriptyline and desipramine). Imipramine also blocks histamine H1 receptors, α1-adrenergic receptors, and muscarinic receptors, which explains their sedative, hypotensive, and anticholinergic effects, respectively [349]. Unspecified indications for its use include chronic and neuropathic pain [350]. Imipramine reduces the levels of TNF-α and IL-1β in primary culture of mixed glial cells of rats stimulated with lipopolysaccharide [351], and it also reduces the level of IL-6 in the plasma of stressed 57BL/6 mice [352]. Imipramine has been shown to reduce IFN-γ and IL-6 levels in the rat hypothalamus [353], but the effect of imipramine on cytokine imbalance in patients with IDD needs further study.

Gabapentin (1-(aminomethyl)cyclohexaneacetic acid) is a structural analogue of the inhibitory neurotransmitter gamma-aminobutyric acid (GABA); this drug belongs to the group of anticonvulsants [38]. Gabapentin has a relatively mild ADR profile, a broad therapeutic index, and no discernible hepatic metabolism. It is structurally and functionally related to another GABA derivative, pregabalin [354]. The main mechanism of action of gabapentin appears to be related to the accessory α2δ-1 subunit of voltage-gated calcium channels [355]. There is evidence that chronic pain can induce an increase in the expression of α2δ subunits, and these changes correlate with hyperalgesia [356]. Gabapentin inhibits the action of α2δ-1 subunits, thereby reducing the density of presynaptic voltage-gated calcium channels and the subsequent release of excitatory neurotransmitters [355], which is also responsible for the antiepileptic and normothymic effects of gabapentin [357]. Overall, these effects may inhibit the overexpression of pro-inflammatory cytokines in response to pain and secondary depressive disorders in patients with IDD and chronic discogenic back pain.

Pregabalin ((S)-3-(aminomethyl)-5-methylhexanoic acid) is structurally similar to GABA and belongs to anticonvulsants [358]. The proposed mechanism of its action is that the drug presynaptically binds to the α2-δ subunit of voltage-gated calcium channels [38] in the central nervous system. Through this mechanism, pregabalin modulates the release of excitatory neurotransmitters (glutamate, substance-P, norepinephrine, and calcitonin gene-related peptide) from moving from the dorsal root ganglia to the dorsal horns of the spinal cord [359], which may also contribute to possess its analgesic and anti-inflammatory effects. Although pregabalin is a structural derivative of the inhibitory neurotransmitter GABA, it does not bind directly to GABA or benzodiazepine receptors [360], providing a good tolerability profile. Calcium channels are activated both in the spinal cord and in the spinal roots and ganglia after surgical trauma, for example in IDD patients after IVD hernia surgery. The analgesic effect may be due to inhibition of calcium influx through these channels. Both gabapentin and pregabalin have shown promising results in reducing neuropathic pain after spinal anesthesia-surgery in men [361]. Lower rates of postoperative pain have been reported in patients undergoing lumbar laminectomy treated with pregabalin 1 h prior to induction compared with patients receiving a placebo [362]. In another study, patients undergoing decompressive spinal surgery showed better visual analog scale pain scores at rest and on motion when treated with pregabalin compared with placebo [363]. Pregabalin is known to have several advantages over gabapentin and has only very limited ADRs [364] and better analgesic activity [354]. Pregabalin may reduce postoperative pain after surgical treatment of IDD [365] and pain-mediated overexpression of pro-inflammatory cytokines.

Several studies have provided evidence that gabapentin has anti-inflammatory properties by reducing stimulus-induced expression of inflammatory mediators [366] or by increasing the expression of mediators with established anti-inflammatory properties [367]. Glial elements, glial satellite cells, and especially macrophages in dorsal spinal ganglia culture produce pro-inflammatory cytokines (TNF-α and IL-6) in the presence of lipopolysaccharide in the culture medium. As a consequence, neurons in the dorsal spinal ganglia exhibit an enhanced response to stimulation with capsaicin [368], a transient receptor potential vanilloid 1 (TRPV-1) channel agonist that has been identified as the most important transducer of noxious stimuli [369]. Most neurons in the dorsal spinal ganglia are nociceptors, and many of them are equipped with the TRPV-1 pain-sensing channel of the transient receptor potential channel family [370]. Dorsal spinal ganglion neurons additionally express L-type Ca2+ channels with their α2δ-1 subunit, which can be easily activated by depolarizing stimuli such as the capsaicin-induced influx of calcium ions. These subunits are targets for gabapentinoids. Two putative mechanisms of action of gabapentinoids on the dorsal spinal ganglia have been described. On the one hand, there are neurons equipped with TRPV-1 and voltage-gated calcium channels [371], responses to which can be attenuated by gabapentinoids. On the other hand, there are glial elements where the production of inflammatory mediators [370] can be regulated by gabapentinoids through mechanisms that have not yet been identified [366,367]. Therefore, lipopolysaccharide-induced expression and overexpression of pro-inflammatory cytokines and other inflammatory mediators is attenuated in primary dorsal spinal ganglia cultures in the presence of gabapentin or pregabalin. In animal models, gabapentinoids have been shown to inhibit the formation of several inflammatory mediators [366], although the results of studies by Leisengang et al. [372] did not indicate a strong anti-inflammatory effect of gabapentin or pregabalin on dorsal spinal ganglia. However, the authors showed the inhibition of lipopolysaccharide-induced expression of IL-6 and COX-2 in the culture medium. IL-6 likely influences peripheral sensory nerves in a way that contributes to the manifestation of inflammatory/neuropathic diseases [373]. As shown by Fang et al. [373], the contribution of IL-6 to the manifestation of acute and chronic pain is mediated by the overexpression of the TRPV-1 capsaicin receptor under the influence of this pro-inflammatory cytokine. At the same time, a trend towards an increase in LPS-induced expression of TRPV-1 was observed only in the absence of gabapentinoids. Li et al. [374] demonstrated that gabapentin also reduces brain tissue damage during intracerebral bleeding by suppressing NF-κB-mediated neuroinflammation in vitro. It also improved behavioral performance, decreased phosphorylated IκB-α and cleaved caspase 3 levels, apoptosis rate, and serum IL-6 levels in rats. Thus, gabapentioids are the most promising drugs from the group of anticonvulsants for the correction of cytokine imbalance in IDD patients with discogenic back pain.

Levetiracetam ((αS)-α-ethyl-2-oxo-1-pyrrolidineacetamide) is an anticonvulsant from the pyrrolidine class [38]. The binding of levetiracetam to the synaptic vesicle protein 2A (SV2A) is a key factor in its mechanism of action. SV2A is known to be a membrane-bound protein found in synaptic vesicles and ubiquitous in the CNS [375]. This protein plays a role in vesicle exocytosis [38] and in the modulation of synaptic transmission by increasing the available number of secretory vesicles available for neurotransmission [376]. Stimulation of presynaptic SV2A by levetiracetam can inhibit the release of excitatory neurotransmitters [377] without inhibiting normal neurotransmission [375]. Levetiracetam has also been shown to indirectly affect GAM-Kergic neurotransmission [38]. In addition, the anti-inflammatory properties of levetiracetam have been described, which have received increasing attention in recent years [378]. Some studies have shown that the anticonvulsant effect of this drug is due to a variety of mechanisms, including the anti-inflammatory effect established by Stienen et al. [379]. The authors hypothesized that this anti-inflammatory effect is mediated by levetiracetam’s inhibition of the expression of pro-inflammatory cytokines (particularly IL-1β, IL-6 and TNF-α). The results of Abed et al. [380] showed that levetiracetam reversed elevated serum levels of IL-1β and IL-6 in an animal model of lipopolysaccharide adjuvant-induced arthritis. In addition, levetiracetam attenuated the upregulation of P-JAK2 and P-STAT3 in the synovium in all arthritis groups. Levetiracetam also had a significant inhibition of both TLR-4 and p-MAPK expression in all treated rats. Levetiracetam can inhibit the JAK-2/STAT3 and TLR-4/MAPK signaling pathways involved in the pathogenesis of arthritis by reducing the negative effects of lipopolysaccharide on cytokine balance and inflammation.

Venfalaxine (1-[2-(dimethylamino)-1-(4-methoxyphenyl)ethyl]cyclohexanol) is a serotonin/norepinephrine reuptake inhibitor. This drug belongs to the group of antidepressants [381]. Vollmar et al. [381] showed that venfalaxine has an anti-inflammatory effect by suppressing the expression of several pro-inflammatory cytokines (e.g., IL-6) in vitro. Other authors have confirmed its immunomodulatory activity by acting on several pro-inflammatory cytokines (e.g., TNF-α) [382]. In a study by Kamel et al. [383] venfalaxine reduced the level of IL-6 in the limb homogenate, which is confirmed by the work of Vollmar et al. [382], who demonstrated a reduced level of IL-6 in murine experimental autoimmune encephalomyelitis after treatment with venfalaxine. This may be due to a decrease in the migration of activated lymphocytes into inflamed animal tissues due to changes in the expression of genes involved in the activation and ion transport of lymphocytes [384]. Venfalaxine suppressed the expression of *STAT3* genes in animals with experimental arthritis [385]. The anti-inflammatory effect of venfalaxine was further emphasized through a significant suppression of the expression of the pro-inflammatory cytokine IL-17 through a decrease in the expression of STAT-3 in Th17 lymphocytes [386]. Additionally, in animals treated with venfalaxine, a marked decrease in the level of NF-κB in tissues was observed [383]. This means that the inhibition of the key pro-inflammatory cytokine IL-17, as well as other inflammatory mediators (TNF-α and IL-1β), as well as reactive oxygen species, is possible with venfalaxine [387]. The inhibition of the expression of pro-inflammatory cytokines and other inflammatory mediators during venlafaxine therapy significantly reduces the severity of arthritis in various animal models [388]. Venfalaxine also caused a marked decrease in MMP-3 and RANKL levels [383]. Preclinical and clinical studies of venlafaxine confirm the prospect of using this drug for the correction of cytokine imbalance in IDD.

### 3.11. Drugs of Other Groups

#### 3.11.1. Suppressive Cytokine Anti-Inflammatory Agents

In the late 1980s, a new class of anti-inflammatory drugs was developed, called cytokine suppressive anti-inflammatory drugs (CSAIDTM). The prototype of this class of compounds is SB203580, a drug with a new mechanism of action [389]. These pyridine imidazole compounds have a potent inhibitory effect on the production of pro-inflammatory cytokines in vitro, and in vivo they can attenuate the inflammatory component of diseases in the absence of generalized immunosuppression. The molecular targets of CSAIDTM have been identified as a pair of closely related MAPK homologues, alternatively referred to as cytokine-suppressive binding protein (CSBP) [390], p38 [391], or RK [392]. p38 MAPK inhibitors have been shown to affect cytokine imbalance in a non-selective manner [393], in contrast to ERK1/2 inhibitors that block TNF-α and IL-1β, but not IL-10 [394].

#### 3.11.2. Renin Inhibitors

Aliskiren ((2S,4S,5S,7S)-5-amino-N-(2-carbamoyl-2-methylpropyl)-4-hydroxy-7-{[4-methoxy-3-(3-methoxypropoxy)phenyl]methyl }-8-methyl-2-propan-2-ylnonanamide) is a renin inhibitor that reduces the level of angiotensin II with a subsequent decrease in the inflammatory response caused by the stimulation of the renin-angiotensin system [395]. Its effectiveness has been proven previously in an experimental model of osteoarthritis due to its inhibitory effect on the renin-angiotensin system present in cartilage, with a subsequent reduction in erosion [396]. Data have been accumulated on the local involvement of the renin-angiotensin system (RAS) in joint diseases [397,398]. Aliskiren has been reported to facilitate bone cell turnover in experimental rats [399]. In addition, the ability of aliskiren to reduce articular cartilage erosion was demonstrated in an experimental rat model of osteoarthritis [396]. Additionally, aliskiren is involved in the regulation of cytokine balance. Thus, the reduction of pro-inflammatory cytokines (TNF-α and IL-6) and biomarkers of oxidative stress with aliskiren was confirmed in an experiment on an animal model [400].

The relationship between the local renin-angiotensin system and the effect of RANKL on bone metabolism was revealed, which was confirmed, for example, by the study by Araújo et al. [401]. Shuai et al. [402] suggested that the local renin-angiotensin system in trabecular bone may contribute to corticoid-induced osteoporosis due to the enhanced effect of RANKL. The above effects of aliskiren may be related to the suppression of intracellular JAK-2/STAT-3 signaling, where an increase in prorenin and its receptor for STAT3 activation has been documented [403]. In addition, various studies have shown that angiotensin II stimulates the JAK-2/STAT-3 pathway [404]. Aliskiren has been shown to have a modulating effect on NOSs, including a decrease in iNOS expression and an increase in eNOS expression, which is consistent with the results of previous studies [405]. The results of these studies provide further clarification of the therapeutic effects of aliskiren against collagen-induced arthritis [316] and explain its potential role in slowing down the development of IDD. However, the efficacy and safety of using aliskiren to correct cytokine imbalance in patients with IDD needs to be clarified in the future.

#### 3.11.3. Acetylcholinesterase Inhibitors

Physostigmine ((3aS-cis)-1,2,3,3a,8,8a-hexahydro-1,3a,8-trimethylpyrrolo[2,3-b]indol-5-ol methylcarbamate) is an inhibitor of acetylcholinesterase, an enzyme responsible for the breakdown of used acetylcholine. By influencing acetylcholine metabolism, physostigmine indirectly stimulates both nicotinic and muscarinic receptors due to a subsequent increase in available acetylcholine at synapses [14]. Acetylcholine appears to play a role in inflammation control as it stimulates the vagus nerve, resulting in reduced cytokine synthesis through the activation of the α7 nicotinic acetylcholine receptors. Conversely, serum acetylcholinesterase activity can have detrimental effects on bone and cartilage erosion. Cholinesterase activity has been demonstrated to increase with age, contributing to the risk and severity of arthritis in the elderly [406].

For example, the use of acetylcholinesterase inhibitors has shown a positive effect in the treatment of rheumatoid arthritis, which is associated with the control of the release of pro-inflammatory cytokines (e.g., TNF-α) and the mitigation of inflammation [407]. Ahsan et al. [408] showed that physostigmine significantly prevented the denaturation of albumin protein from hen egg and bovine serum sources in a dose-dependent manner and it also had a significant stabilizing effect on the erythrocyte membrane at higher concentrations, which may be due to the effect of removing free radicals or with the inhibition of the release of intracellular components and inflammatory mediators. In addition, physostigmine caused a significant decrease in serum levels of prostaglandin E2 and pro-inflammatory cytokine TNF-α.

#### 3.11.4. Xanthines

Theophylline (1,3-dimethylxanthine) is a smooth muscle relaxant, cardiac and central nervous system stimulant. Theophylline acts as a phosphodiesterase inhibitor, adenosine receptor blocker, and histone deacetylase activator [14].

Theophylline may be a promising agent for the treatment of immunological inflammatory conditions, including the correction of cytokine imbalance in IDD. Liang et al. [409] reported an immunomodulatory effect of theophylline in an animal model. Theophylline modulates cytokine status and NO synthesis [410]. Ghasemi-Pirbaluti et al. [411] demonstrated that this drug can modulate myeloperoxidase and the production of pro-inflammatory cytokines. It has been demonstrated that theophylline has a good anti-inflammatory effect due to the activation of histone deacetylases, as well as the modulation of MMPs and other inflammatory mediators [412]. Additionally, it can modulate JAK/STAT/RANKL cytokine signaling in animals treated with complete Freund’s adjuvant, growth hormone, or a combination of the two. Thus, physostigmine plays an important role in the activation and differentiation of osteoclasts [413].

#### 3.11.5. Nitrates

Nicorandil (2-(pyridine-3-carbonylamino)ethyl nitrate) is an effective oral vasodilator and antianginal agent that causes the vasodilation of arterioles and large coronary arteries by activating potassium channels [14].

It has been reported that nicorandil promotes the opening of potassium channels and has anti-inflammatory and immunomodulatory potential against inflammation in the experiment [414]. Nicorandil was found to prevent ulcerogen-induced TNF-α production [415]. The results of Gaafar et al. [413] demonstrated that this drug significantly inhibited JAK/STAT/RANKL/cytokine pathway signaling. It has immunomodulatory and anti-inflammatory effects mediated by the modulation of this signaling pathway [416]. According to Zhao et al. [417], substances that open potassium channels can protect in vitro against neuroinflammation caused by oxygen/glucose deficiency by inhibiting the activation of pro-inflammatory cytokines and the signal transduction of the Toll-like receptor 4.

#### 3.11.6. Direct Oral Anticoagulants

Apixaban (1-(4-methoxyphenyl)-7-oxo-6-[4-(2-oxo-piperidin-1-yl)phenyl]-4,5-dihydropyrazolo[5,4-c]pyridine-3-carboxamide) is a potent, selective, reversible direct inhibitor of Hageman factor (FXa); it is used to prevent venous thromboembolism [418]. In addition, apixaban has powerful anti-inflammatory and remodeling effects on many diseases. These effects can be explained by the inhibition of the poly-ADP ribose polymerase (PARP) enzyme on extravascular cells [419].

Previously, a significant correlation has been established between the activation of blood coagulation factors and the stimulation of various inflammatory pathways [420]. Anticoagulant therapy not only affects the coagulation cascade but can also inhibit inflammatory mediators (including pro-inflammatory cytokines), indicating a wide interaction between these processes [421]. It has been shown that FXa is an effective inducer of kinase phosphorylation, which is associated with a significant increase in the level of P-JAK2, P-STAT3, and P-MAPK in synovial tissue. Additionally, an increase in P-JAK2, P-STAT3, and P-MAPK levels was observed when Freud’s complete adjuvant group of experimental animals were stimulated with FXa. Apixaban significantly interfered with the expression of P-JAK2, P-STAT3, and P-MAPK in the synovium. The inhibition of FXa by apix-aban promoted the potent inhibition of JAK2/STAT3 and MAPK-activated pathways. It has been shown that the activation of FXa coagulation induces mRNA expression of the PAR-2 gene [422]. Bae et al. [423] reported that FXa exerts its action through the PAR-2 receptor. Consistent with the findings of Hollborn et al. [424] and Senden et al. [425], the results of El-Ghafar et al. [422] showed a significant increase in the levels of platelet growth factor and the pro-inflammatory cytokine IL-6 after FXa activation. Tissue PAR-2 levels, serum levels of platelet growth factor, and IL-6 levels were significantly reduced in all apixaban-treated rats compared to normal controls. Apixaban had a lower inhibitory effect on IL-6 compared to the other parameters listed above. This can be explained by its main action as a selective inhibitor of FXa, which subsequently leads to a significant inhibition of FXa-activated parameters such as PAR-2, platelet growth factor, and (to a lesser extent) IL-6, since the increase in the level of IL-6 expression is dependent on various signals and does not depend solely on FXa activation [426]. However, FXa-induced JAK2/STAT3 and MAPK phosphorylation may be directly related to platelet growth factor and IL-6 activation [427]. This effect may be indirectly related to PAR-2 activation, which mediates kinase and tyrosine phosphorylation, including the JAK, STAT, and MAPK pathways [428,429].

## 4. Discussion

**Figure 3:**
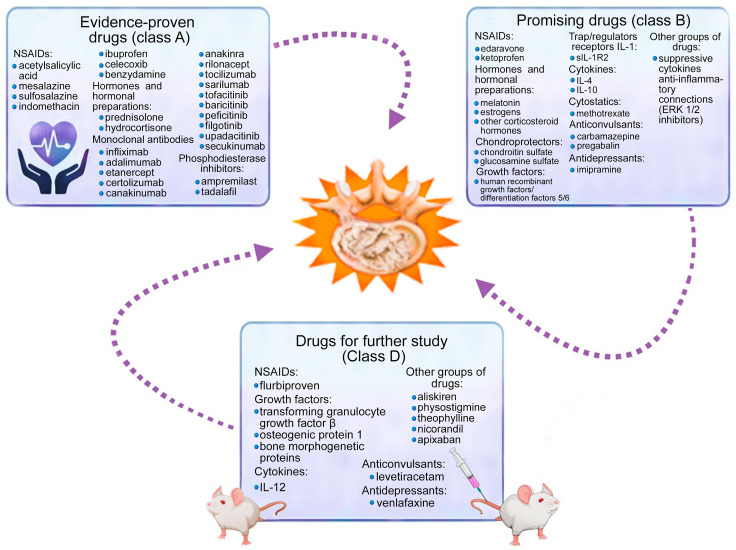
Drugs for correction of cytokine imbalance in intervertebral disc degeneration.

This narrative review suggests that drugs of various pharmacological groups are used or can be used in the future to correct cytokine imbalance in patients with IDD [6] and chronic discogenic back pain (Figure 3).

**Figure 4:**
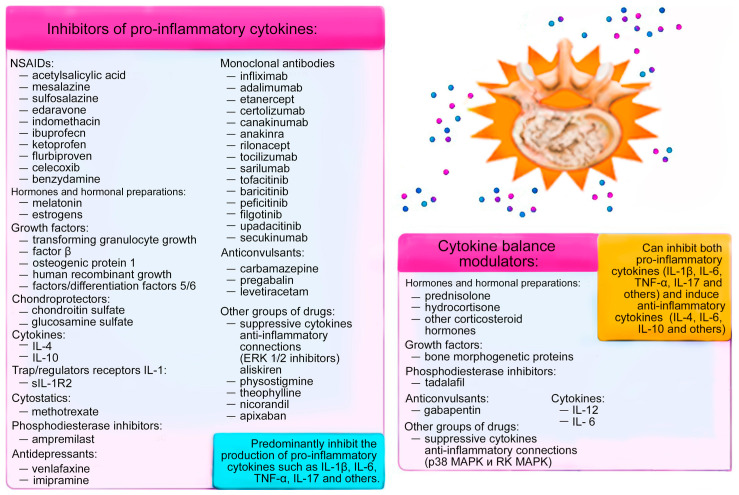
Inhibitors and modulators of pro-inflammatory cytokines in patients with intervertebral disc degeneration.

These prospects are based on the results of studies of the molecular mechanisms of development and persistence of cytokine imbalance during the development of IDD, especially in severe disease, as well as the results of preclinical (experimental) and clinical studies of the molecular mechanisms of action of drugs that modulate the cytokine balance, including the inhibition of the level and/or effects of pro-inflammatory cytokines or stimulation of anti-inflammatory cytokines (Table 1). The various molecular mechanisms of action of the drugs discussed in this review may be involved in maintaining an optimal balance between pro-inflammatory and anti-inflammatory cytokine levels, which determines their overall effect on inflammatory responses in patients with IDD (Figure 4). Pharmacotherapy of the cytokine imbalance can direct the protective immune response in the IDD patient towards the reduction of chronic inflammation (anti-inflammatory effect) or towards healing (regenerative effect). Thus, understanding the molecular mechanisms of drugs to cytokine imbalance may be useful for choosing an effective and safe treatment for patients with IDD by initiating anti-inflammatory and regenerative responses in NP and AF cells, as well as in the extracellular matrix in IVDs. However, the over- or under-inhibition of pro-inflammatory cytokines, as well as under- or over-stimulation of anti-inflammatory cytokines, can be detrimental and trigger the development of ADRs in patients with IDD.

To date, therapeutic strategies targeting pro-inflammatory cytokines may be effective in the treatment of IDD and severe discogenic back pain. These therapeutic strategies are based on the fact that pro-inflammatory cytokines are critical for initiating an inflammatory response in degenerating IVDs, facet joints, and surrounding tissues. However, levels of pro-inflammatory cytokines in degenerating IVDs may reach their absolute or relative peak before the clinical signs of IDD become apparent. In addition, therapeutic strategies aimed at the blockade of pro-inflammatory cytokines, paradoxically, can lead to an increase in the induced process to increase inflammation in some patients with IDD [6,430].

This descriptive review provides new insight into the known and promising molecular mechanisms of action of drugs to correct cytokine imbalances in patients with IDD. On the one hand, this knowledge can help practicing neurologists and orthopedists improve the efficacy and safety of treating IDD and discogenic back pain that are resistant to previously used classical drug and non-drug (physiotherapy, surgical) strategies. On the other hand, this knowledge may help pharmacologists and pharmacologists to develop new drugs to correct cytokine imbalances in IDD and severe discogenic back pain.

## 5. Conclusions

This review demonstrates that the problem of correcting cytokine imbalance in patients with IDD is far from being resolved. However, the results of studies on the molecular mechanisms of the influence of previously known and new drugs on pro-inflammatory and anti-inflammatory cytokines in degenerating IVDs, facet joints, and surrounding tissues of the spine may help answer previously unresolved questions about the possibilities of achieving the expected therapeutic response in patients with IDD and back pain. In addition, the inconsistency of the results of some previous studies on the efficacy and safety of drugs that modulate the levels and/or effects of pro-inflammatory and anti-inflammatory cytokines, as well as their signaling pathways, indicates that new personalized therapeutic strategies for correcting cytokine imbalance in IDD are likely to lead to the need to revise existing approaches to the treatment of this disease in the future. In general, it should be based not on the study of the serum level of one cytokine, but on the assessment of the balance between non-inflammatory and anti-inflammatory cytokines in a particular patient. This is supported by the previously formulated hypothesis that cytokine imbalance may be an important biomarker of acute and chronic inflammatory responses in patients with IDD.

## Figures and Tables

**Table 1:** Drugs for correction of cytokine imbalance in intervertebral disc degeneration.

Drug	Evidence Class	References
A. Nonsteroidal Anti-Inflammatory Drugs		
Acetylsalicylic acid	A	[15,16,17,18,19,20,21,22]
Mesalazine	A	[23,24]
	A	[25]
Sulfasalazine	A	[23,28]
Edaravone	B	[29]
Indomethacin	A	[31,32]
	B	[31,32]
Ibuprofen	A	[36]
Ketoprofen	B	[36,37]
Flurbiprofen	D	[36]
Celecoxib	A	[41,42]
	B	[41,42]
Benzydamine	A	[44,45]
	B	[46]
B. Hormones		
Prednisolone	A	[53,62]
Hydrocortisone	A	[63,65,66]
	B	[64]
Corticosteroids	A	[53,54]
	B	[55,242]
Melatonin	B	[68,69,70]
	D	[71,72,73,74]
Estrogens	B	[83,86,94,98]
	D	[95]
C. Growth Factors		
Bone morphogenetic proteins	D	[105,106,107]
Transforming growth factor beta	D	[115,116,118]
	B	[127]
Insulin-like growth factors	D	[162,163,164,165,166,167,168,283]
Osteogenic protein 1	D	[173,174]
Human recombinant growth factors/differentiation 5/6	B	[194,195,196,197,198,199]
D. Chondroprotectors		
Chondroitin sulfate	B	[203,206,207,208,209,211]
Glucosamine sulfate	B	[214,215,216,217,219]
E. Trap Receptors for Pro-inflammatory Cytokines		
sIL-1R2	B	[221,265]
F. Interleukins Regulating Pro-Inflammatory Cytokines		
Interleukin 4	B	[53,227,228,229,236]
Interleukin 10	B	[231,232]
Interleukin 12	D	[245]
	B	[234,246]
G. Monoclonal Antibodies		
Infliximab (antibodies against tumor necrosis factor α)	A	[251]
	B	[66,252]
Adalimumab (antibodies against tumor necrosis factor α)	A	[38,254]
Etanercept (antibodies against tumor necrosis factor α)	A	[258]
Certolizumab (antibodies against tumor necrosis factor α)	A	[259]
Canakinumab, anakinra, rilonacept (antibodies against interleukin 1 α/β)	A	[247]
Tocilizumab (antibodies against interleukin 6)	A	[14]
Sarilumab (antibodies against interleukin 6)	A	[278]
	B	[279,280]
Tofacitinib (antibodies against Janus kinase)	A	[14,283]
	B	[282]
Baricitinib (antibodies against Janus kinase)	A	[38]
	B	[287]
Peficitinib (antibodies against Janus kinase)	A	[288]
Filgotinib (antibodies against Janus kinase)	A	[289]
	B	[290]
Upadacitinib (antibodies against Janus kinase)	A	[38]
Secukinumab, anakinra, rilonacept (antibodies against interleukin 17)	A	[14,302,306]
	B	[305]
H. Cytostatics		
Methotrexate	B	[311,312,313,315,316]
I. Phosphodiesterase Inhibitors		
Apremilast	A	[319]
	B	[320,322,323]
Tadalafil	A	[325,328,330]
	B	[316,327,331]
J. Anticonvulsants		
Carbamazepine	B	[351,355]
Gabapentin	B	[368,369,375]
	A	[357,358,363]
Pregabalin	B	[375]
	A	[361,363,364]
Levetiracetam	B	[381,382]
K. Antidepressants		
Venfalaxine	B	[383,384,385,387,388,389]
Imipramine	B	[350,351,353,354,355]
L. Other		
Pyridine imidazole	B	[392,393,395,396]
Aliskiren	B	[403,404,405,406,407]
Physostigmine	B	[408,409,410]
Theophylline	B	[412,413,414,415]
Nicorandil	B	[415,417,418]
Apixaban	B	[424,426,427,428,429]

## Data Availability

Not applicable.
